# Leveraging Guided Backpropagation to Select Convolutional Neural Networks for Plant Classification

**DOI:** 10.3389/frai.2022.871162

**Published:** 2022-05-11

**Authors:** Sakib Mostafa, Debajyoti Mondal, Michael A. Beck, Christopher P. Bidinosti, Christopher J. Henry, Ian Stavness

**Affiliations:** ^1^Department of Computer Science, University of Saskatchewan, Saskatoon, SK, Canada; ^2^Department of Physics, University of Winnipeg, Winnipeg, MB, Canada; ^3^Department of Applied Science, University of Winnipeg, Winnipeg, MB, Canada

**Keywords:** explainable AI, deep learning—artificial neural network, Guided Backpropagation, neural network visualization, convolutional neural network

## Abstract

The development of state-of-the-art convolutional neural networks (CNN) has allowed researchers to perform plant classification tasks previously thought impossible and rely on human judgment. Researchers often develop complex CNN models to achieve better performances, introducing over-parameterization and forcing the model to overfit on a training dataset. The most popular process for evaluating overfitting in a deep learning model is using accuracy and loss curves. Train and loss curves may help understand the performance of a model but do not provide guidance on how the model could be modified to attain better performance. In this article, we analyzed the relation between the features learned by a model and its capacity and showed that a model with higher representational capacity might learn many subtle features that may negatively affect its performance. Next, we showed that the shallow layers of a deep learning model learn more diverse features than the ones learned by the deeper layers. Finally, we propose SSIM cut curve, a new way to select the depth of a CNN model by using the pairwise similarity matrix between the visualization of the features learned at different depths by using Guided Backpropagation. We showed that our proposed method could potentially pave a new way to select a better CNN model.

## 1. Introduction

Deep learning approaches have been widely adopted into agriculture (Weng et al., 2019; Chandra et al., 2020) (i.e., precision agriculture, crop breeding, plant phenotyping) due to their ability to extract complex features from a large amount of data (Montavon et al., 2019). In recent years, the focus has shifted toward developing tools to optimize the performance of the models to help researchers integrate deep learning models easily into their studies (Humphrey et al., 2017; Ubbens and Stavness, 2017; Ubbens et al., 2018). Despite the recent development, deep learning models are often considered as “black box” (Tzeng and Ma, 2005; Oh et al., 2019). To improve the trustworthiness of models and to design them effectively for the unique challenges that appear with specialized datasets, many recent studies have focused on explaining the learning and prediction of deep learning models (Tzeng and Ma, 2005; Mostafa and Mondal, 2021). However, explainable deep learning models in plant phenotyping still remains to be an active field of research with room for improvement (Ubbens and Stavness, 2017; Chandra et al., 2020; Hati and Singh, 2021). Plant image datasets are often different from general image datasets due to small sample sizes, highly self-similar foreground objects, and simplified backgrounds. Therefore, complex deep learning models that are used for general image classification may perform poorly for plant datasets (Mohanty et al., 2016; Zenkl et al., 2022).

Convolutional neural networks (CNN) are one of the most widely used deep learning models in image-based plant phenotyping. A common phenomenon when designing a CNN model is model overfitting. Overfitting in a CNN model occurs when the model approximates or memorizes the training data and fails to generalize to unseen examples in the testing data (Reed and Marks, 1999). A popular way to detect the overfitting is by inspecting the difference between the training and testing accuracy and loss using the accuracy and loss curve (Géron, 2019; Gigante et al., 2019). However, this does not provide insight into the model's learning or which features or part of the image contributed to the model's prediction.

### 1.1. Explainability in CNN

To explain the learning of CNN models, researchers have proposed different feature-map visualization techniques (Springenberg et al., 2014; Bach et al., 2015; Ribeiro et al., 2016; Selvaraju et al., 2016; Lundberg and Lee, 2017). Zeiler and Fergus (2014) proposed deconvolutional networks (Deconvnet) that provide insight into the function of a CNN classifier's intermediate layers by modifying the model's gradient and displaying the visual patterns in the input image that generated the activation. There have been several attempts that deviate from deconvolutional networks. Simonyan et al. (2013) used the gradient of a CNN model's output with respect to the input image's pixel intensities to generate saliency maps. Zhou et al. (2016) and Selvaraju et al. (2017) proposed class activation mapping (CAM), and Gradient-weighted Class Activation Mapping (Grad-CAM), respectively, which helps achieve class-specific feature visualization.

Ghosal et al. (2018) visualized feature maps in various layers that detected the stress regions of a plant leaf. Nagasubramanian et al. (2019) used a saliency map based visualization technique to detect the hyperspectral wavelengths that are responsible for the models' performance. Dobrescu et al. (2017) showed that the model always looks at the leaves in the image in the CNN-based plant classifier. In Dobrescu et al. (2019), the research group used layerwise relevance propagation and GBP to explain the learning of intermediate layers of the CNN model by counting the leaves in an image. Escorcia et al. (2015) studied the visualization of the leaf features and found the existence of attribute-centric nodes, which, rather than learning attributes, learns to detect objects. A more recent work, Lu et al. (2021) used guided upsampling and background suppression to improve models' performance. However, their explanation was limited to the visualization of the instances responsible for the count.

Toneva et al. (2018) explained the learning of the CNN models in terms of forgetting patterns, where at some point during the training, the model correctly predicts an example, but eventually, it is misclassified. Feldman (2020) took a different approach and demonstrated that when there are numerous instances of rare examples in the dataset, the deep learning models must memorize the labels to achieve state-of-the-art performance. Feldman and Zhang (2020) showed that along with memorizing outliers, the deep learning models also memorize training examples and if there are testing examples similar to it and hence overparameterized models perform extraordinarily. Salman and Liu (2019) claimed that overfitting is caused due to the continuous update of a deep learning model's gradient and scale sensitiveness of the loss function. They also proposed a consensus-based classification algorithm for limited training examples.

### 1.2. Contributions

In this study, we focus on the plant species classification, which is relevant in digital agriculture, e.g., precision herbicide application (Weis et al., 2008), and is a prevalent task for employing CNN models (Dyrmann et al., 2016; Azlah et al., 2019). We examine the features learned by the intermediate layers of CNN classifiers to understand the behavior of overfit models and the contribution of image background in overfitting. To examine how the CNN models learn in various conditions (overfit or balanced), we use Guided Backpropagation (GBP) (Springenberg et al., 2014) to visualize the features being learned at different layers of the CNN models. We explore whether the GBP-based feature visualizations could be leveraged to detect the overfitting. We then propose a new technique for model selection that can be used to develop balanced models.

There are three main contributions of this study. First, we visualize the intermediate layers of different CNN models to investigate whether the learning of the features depends on the model's capacity. Second, we propose a novel SSIM-based evaluation technique that relates overfitting to the depth of the model and provides an intuitive way to understand the differences between overfit and balanced models. Here SSIM refers to a measurement of the similarity between two feature map visualizations. Third, we show how our SSIM-based evaluation may help detect potential underfitting or overfitting in the CNN models and allow us to select a balanced model (i.e., a model which is neither overfit nor underfit). In particular, it may suffice to examine models of various depths only at their first training epochs, and the corresponding SSIM-based evaluation may reveal a potential balanced model. This approach can reduce the model selection time by several factors compared to the time needed to train different models to select a preferable depth.

## 2. Methodology

### 2.1. Guided Backpropagation

**Figure 1:**
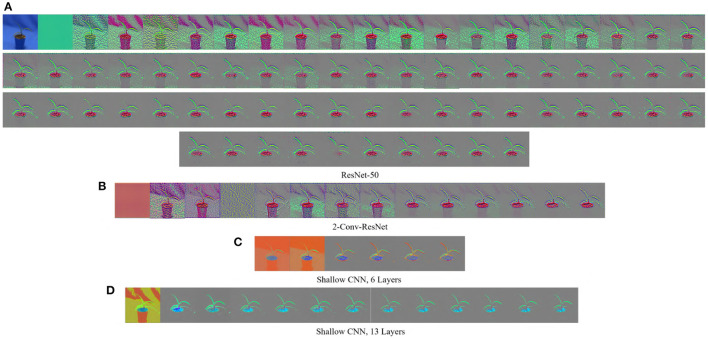
GBP-based visualization of the intermediate layers (left to right) of different CNN models for Barnyard Grass of the Weedling dataset. The top-left image of **(A)** is the input image for all models. **(A)** ResNet-50. **(B)** 2-Conv-ResNet. **(C)** Shallow CNN, 6 layers. **(D)** Shallow CNN, 13 layers.

The GBP is a gradient-based visualization technique that visualizes the gradient with respect to images when backpropagating through the Relu activation function (Springenberg et al., 2014). GBP allows the flow of only the positive gradients by changing the negative gradient values to zero. This allows visualizing the image features that activate the neurons. Let *f* be the feature map of any layer *l* then the forward pass is $f_{i}^{l+1}=Relu\left(f_{i}^{l},0\right)$. Since GBP only allows the flow of positive gradients, the backward pass of the GBP is $R_{i}^{l}=\left(f_{i}^{l}>0\right)\cdot \left(R_{i}^{l+1}>0\right)\cdot \left(R_{i}^{l+1}\right)$, where *R* is an intermediate result on the calculation of the backpropagation for layer *l*. The final output of the GBP is an image of the same dimension as the input, displaying the features of the input image that maximized the activation of the feature maps. A major advantage of GBP is that it works for both convolutional layers and fully connected layers. Figure 1 shows some examples of the visualization generated by GBP for the Weedling dataset using ResNet-50 (He et al., 2016). The gray color in the output of the GBP images (Figure 1) represents that the features in those positions of the input image do not contribute to the prediction.

### 2.2. SSIM Cut Curve

**Figure 2:**
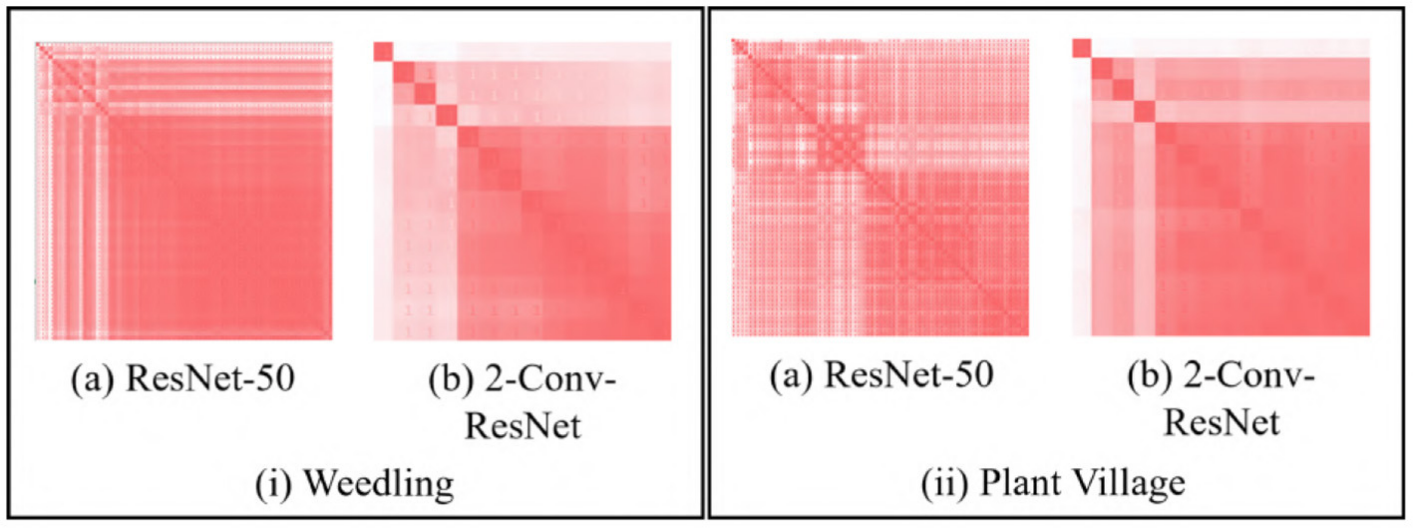
SSIM matrix (*s*_*i, j*_) generated with GBP images for **(i)** Barnyard Grass of the Weedling dataset **(ii)** Apple leaf of the Plant Village dataset using (a) ResNet-50 and (b) 2-Conv-ResNet. A darker red indicates higher SSIM.

We use the GBP approach to visualize the features learned by the intermediate layers of a CNN (e.g., see Figure 1). GBP creates an RGB image with the same shape as the input image representing the learned features for every layer. Figure 2 depicts pairwise SSIM matrices for ResNet-50 and 2-Conv-ResNet models on different datasets, i.e., each entry (*i, j*) denotes the SSIM value between the GBP visualizations obtained for the *i*th and *j*th convolutional layer of ResNet-50 and 2-Conv-ResNet. Here a darker red indicates higher SSIM. From the color-coding, we can observe that the pairwise SSIM is much lower at the initial layers compared to the layers at a deeper layer. This inspired us to find a way to separate the initial (dissimilar) layers from the later (similar) layers. Let *L*_1_, *L*_2_, …, *L*_*n*_ be the GBP visualization for different convolutional layers of a CNN model with *n* convolutional layers. The intuition is that the number *k*, where 1 ≤ *k* ≤ *n*, with the best separation between {*L*_1_, …, *L*_*k*_} and {*L*_*k*+1_, …, *L*_*n*_} would suggest a reasonable depth for the model to have a good performance.

Given a number *k* (i.e., a cut position), we first define a *SSIM cut value* $C_{k}$ to obtain an estimation of how good the cut is for the value *k*. We define $C_{k}$ to be the mean pairwise similarity between {*L*_1_, …, *L*_*k*_} and {*L*_*k*+1_, …, *L*_*n*_}:


(1) $$\begin{array}{r} C_{k}=\frac{1}{k\left(n-k\right)}\overset{k}{\underset{i=1}{\sum }}\overset{n}{\underset{j=k+1}{\sum }}s_{i,j} \end{array}$$

where *s*_*i, j*_ is the SSIM between *L*_*i*_ and *L*_*j*_. In the rest of the article, we will refer to the function $C_{k}$ with respect to *k* as the *SSIM cut curve*.

We can observe this phenomenon better by examining the rate of change, as follows. Let *M*_*i*_ be the sum of the SSIM values of *L*_*i*_ with all other layers. Then $C_{k}$ can be rewritten as $C_{k}=\frac{1}{k\left(n-k\right)}\left(\overset{k}{\underset{i=1}{\sum }}M_{i}-\overset{k}{\underset{i=1}{\sum }}\overset{k}{\underset{j=1}{\sum }}s_{i,j}\right)$. If the curve appears to be flat around the middle cut positions, i.e., when *k*≈(*n*−*k*), then $\Delta C_{k}=C_{k+1}-C_{k}=0$. In other words, we will have $\Delta C_{k}\approx M_{k+1}-2\overset{k}{\underset{i=1}{\sum }}s_{i,k+1}=0$, and hence $\overset{k+1}{\underset{i=1}{\sum }}s_{i,k+1}=\frac{1}{2}M_{k+1}.$ Thus the similarity of *L*_*k*+1_ with the earlier layers {*L*_1_, …, *L*_*k*_} will be equal to its similarity with the rest of the layers {*L*_*k*+1_, …, *L*_*n*_}.

### 2.3. Research Questions

In a CNN, it is expected that the convolutional layers will learn features from the foreground objects in images that are being classified (Kamal et al., 2021). The background features are considered irrelevant, and often these features are not consistent in the images. However, the features learned by a CNN model are also dictated by the model's capacity. Models with a large number of layers have a very high representational capacity, and therefore such models are expected to learn diverse features. Although this is widely believed, no formal exploration has been done in the plant phenotyping context. We thus explore the following research question.

**RQ1:** Are the variety of features learned by a model influenced by the model's capacity?

In a model with high representational capacity, the presence of the potential redundant layers can cause the model to overfit by memorizing irrelevant features. As a result, it performs well for the training images but fails to classify the testing images due to the absence of the features present in the training set. To investigate the presence of redundant layers in CNN models, we considered the following question.

**RQ2:** How diverse are the features learned at different depths in a deep CNN model?

The visualizations of the feature maps represent the learning of the layers. The SSIM similarity of these visualizations may be an effective tool to investigate various options for model depth and potentially select a balanced model.

**RQ3:** Can the SSIM-based evaluation of the feature map visualizations be leveraged to select the required depth of a model?

### 2.4. Datasets

The use of deep learning in plant phenotypic tasks are gradually gaining popularity (Scharr et al., 2016; Aich and Stavness, 2017; Ubbens and Stavness, 2017; Aich et al., 2018), and the dataset plays a vital role as it contains a large amount of noise representing the real-world scenarios. Manually measuring the plant traits is a time-consuming process, which is also prone to error. Image-based automated plant trait analysis using deep learning can help overcome these drawbacks (Aich et al., 2018). However, most of the studies explaining the deep learning models use benchmark datasets (e.g., MNIST (Deng, 2012), Fashion-MNIST (Xiao et al., 2017), and so on), and very few studies have attempted to explain the learning using a plant dataset (Dobrescu et al., 2019).

We used three plant datasets: Weedling dataset (Beck et al., 2020, 2021), Plant Village dataset (Mohanty, 2018), and Plant Seedling dataset (Giselsson et al., 2017) which are commonly used for creating deep learning models for plant phenotyping tasks. For all the datasets, 80% of the available images were used for training and 10% for testing and 10% for validation. The detailed overview of the datasets is available in Mostafa et al. (2021).

### 2.5. Deep Learning Models

**ResNet-50:** In this study, we used the ResNet-50 model with random weight initialization and adam optimizer as the optimization function. We also replaced the top layer of the model with a fully connected layer, where Softmax was the activation function, and the number of neurons was the number of classes in a dataset. We trained the model for 100 epochs and only used the model with the highest testing accuracy.

**2-Conv-ResNet, 3-Conv-ResNet, 4-Conv-ResNet:** Keras ResNet-50 model is an implementation of the architecture proposed by He et al. (2016), where the authors used five convolutional blocks. However, we also used smaller versions of the ResNet-50, where we sequentially increased the number of blocks to create 2-Conv-ResNet, 3-Conv-ResNet, and 4-Conv-ResNet. For example, in 2-Conv-ResNet we only used the layers in Conv1 and Conv2_x (see Table 1, He et al., 2016), and in 3-Conv-ResNet we used the layers in Conv1, Conv2_x, and Conv3_x. In different models, apart from discarding the convolutional blocks, the rest of the architecture remained the same. We used the modified ResNet models to investigate the relation between the model depth and SSIM cut curve and to see whether decreasing the depth helps avoid overfitting.

**Table 1:** Performances of different models for various datasets.

**Dataset name**	**Model name**	**Training accuracy (%)**	**Testing accuracy (%)**	**Validation accuracy (%)**
Weedling	ResNet-50	98.70	96.70	96.29
	ResNet-50-10%	99.89	50.70	62.88
	2-Conv-ResNet	99.88	95.53	95.62
	2-Conv-ResNet-10%	99.89	52.10	44.68
	3-Conv-ResNet	99.93	95.14	95.13
	4-Conv-ResNet	99.75	94.21	94.41
	Shallow CNN, 6 Layers	94.00	89.60	88.73
	Shallow CNN, 13 Layers	96.23	95.45	94.91
Plant	ResNet-50	98.59	98.04	98.27
village	ResNet-50-10%	87.99	77.93	65.45
	2-Conv-ResNet	99.25	99.17	99.31
	2-Conv-ResNet-10%	90.91	82.57	65.02
	3-Conv-ResNet	99.65	99.29	99.30
	4-Conv-ResNet	96.74	96.91	96.83
	Shallow CNN, 6 Layers	98.26	96.46	97.75
	Shallow CNN, 13 Layers	96.96	96.46	96.48
Plant	ResNet-50	91.26	81.90	80.38
seedling	2-Conv-ResNet	85.16	68.75	61.67
	Shallow CNN, 6 Layers	90.51	76.79	68.79
	Shallow CNN, 13 Layers	68.41	69.22	64.54

*Bold values represent the highest classification accuracy*.

**ResNet-50-10% and 2-Conv-ResNet-10%:** In an attempt to create overfit models for this study, we trained the ResNet-50 and 2-Conv-ResNet on 10% training data for the Weedling and Plant Village dataset; but we left out the Plant Seedling dataset due to its small size.

**Shallow CNN:** Along with the ResNet-50, we also used two shallow CNN models for our experiments: one with 6 convolutional layers and the other with 13 convolutional layers, which we named **Shallow CNN, 6 Layers** and **Shallow CNN, 13 Layers**, respectively. In the shallow CNN models, we only used a combination of convolutional layers and avoided using the residual connection. These models aim to examine whether the observations obtained from the comparative analysis between ResNet-50 and 2-Conv-ResNet also hold for shallow CNN models.

For the shallow CNNs, we used categorical cross-entropy as the loss function, random weight initialization, and adam optimizer to optimize the models. Similar to ResNet models, we trained shallow models for 100 epochs with a minibatch size of 16 and chose the model with maximum testing accuracy. While training the shallow CNNs on the Weedling dataset, we resized the images to 512 × 512. For the other datasets, the size of the images was 224 × 224, as it is required for the ResNet models. We used varying zoom range, image flipping, and distorting images along an axis (shear angle) for data augmentation and added an additional batch of augmented images during each epoch. The model architecture and more details of the shallow CNN models are in the Supplementary Material.

The training, testing, and validation accuracy of different models on the different datasets are in Table 1. In this study, for the Weedling and Plant village dataset, we have considered the ResNet-50-10% and 2-Conv-ResNet-10% as overfit models due to their significant difference between training accuracy and testing accuracy. All the models for the Plant Seedling dataset were overfit except the shallow CNN with 13 convolutional layers, which had a very poor accuracy indicating the model was not optimized for the classification.

## 3. Result and Discussion

### 3.1. Learning of Intermediate Layers

A CNN model is expected to extract features from the foreground of the images (Xiao et al., 2020). The foreground of an image is the object that we are performing the task on. Hence we first examined GBP visualization of the features being learned by the intermediate layers in various models.

**Figure 3:**
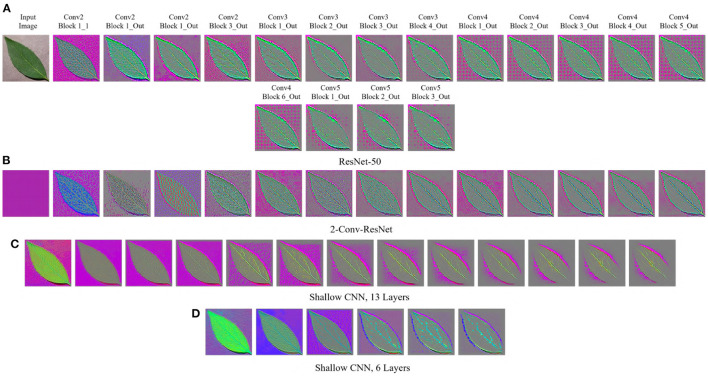
GBP-based visualization of the intermediate layers (left to right) of different CNN models for Bean leaf of the Plant Village dataset. The top-left image of **(A)** is the input image for all models. **(A)** ResNet-50. **(B)** 2-Conv-ResNet. **(C)** Shallow CNN, 13 layers. **(D)** Shallow CNN, 6 layers.

Figures 1, 3 show the GBP visualization of the consecutive layers of different models for the Weedling and Plant Village datasets, respectively. After inspecting the visualized features, we can see that in Figure 1 the last convolutional layer of ResNet models (ResNet-50 and 2-Conv-ResNet) extracted features from the plant leaf, soil, and plant pot (zoom the figure for a better view) based on which the classification is performed. There is also the influence of background features on the ResNet models, although it is not very strong. On the other hand, the shallow models (Shallow CNN, 6 Layers and Shallow CNN, 13 Layers) learned features from the plant leaf and soil. There is no visible influence of the background features.

For the Plant Village dataset (Figure 3), the features extracted by both ResNet models are strongly influenced by the background of the image. The background of the Plant Village dataset consisted of grainy structures, which might have forced the ResNet models to extract features from them. However, the shallow CNN models only extracted features from the leaf edge. If we closely inspect the visualized features, we can see that the shallow models also extracted features from the leaf's veins. In contrast, the ResNet models also depended on the leaf pixels. Between the ResNet models, the ResNet-50 extracted more features from the image background than the 2-Conv-ResNet. This observation is consistent for both datasets.

Analyzing Figures 1, 3, one can observe that the variety of features learned by a model depends on the capacity of the model. ResNet-50 has the highest representational capacity, followed by the 2-Conv-ResNet, Shallow CNN, 13 Layers, and Shallow CNN, 6 Layers. The figures show that the variety of extracted features was higher for the ResNet-50 than other models. Although the background seems uniform in the Weedling dataset, the lighting condition varied for different images. The presence of irregular bright patches might have been deemed as a feature to the ResNet-50 model, which it learned due to its higher representational capacity. The extraction of such features decreased with the decrease of the model capacity. The same trend was followed by the models used for the Plant Village dataset. The analysis of Figures 1, 3 reveal that the features learned by a model is influenced by the model's capacity (**RQ1**).

### 3.2. Contribution of Model Depth to Performance

When designing a CNN model, a common practice is to increase the depth of the model to achieve better performance. In this section, we studied whether increasing the depth of the model helps learn better features. From Figures 1, 3, we can see that the ResNet-50 models have the highest number of convolutional layers. Examining the features extracted by the convolutional layers, it is evident that for the ResNet-50 model after a certain depth, GBP visualizations are similar. However, for the 2-Conv-ResNet, and both shallow models, the GBP visualizations were dissimilar across all the layers. To quantify the similarity of features extracted by different layers, we propose SSIM cut curve. For every class in a dataset, we randomly selected an image from the testing set and calculated the SSIM cut values for the images (see Section 2.2). Next, we averaged the SSIM cut values over all the images for every layer of a CNN model. Thus for every model, we ended up with an SSIM cut curve.

**Figure 4:**
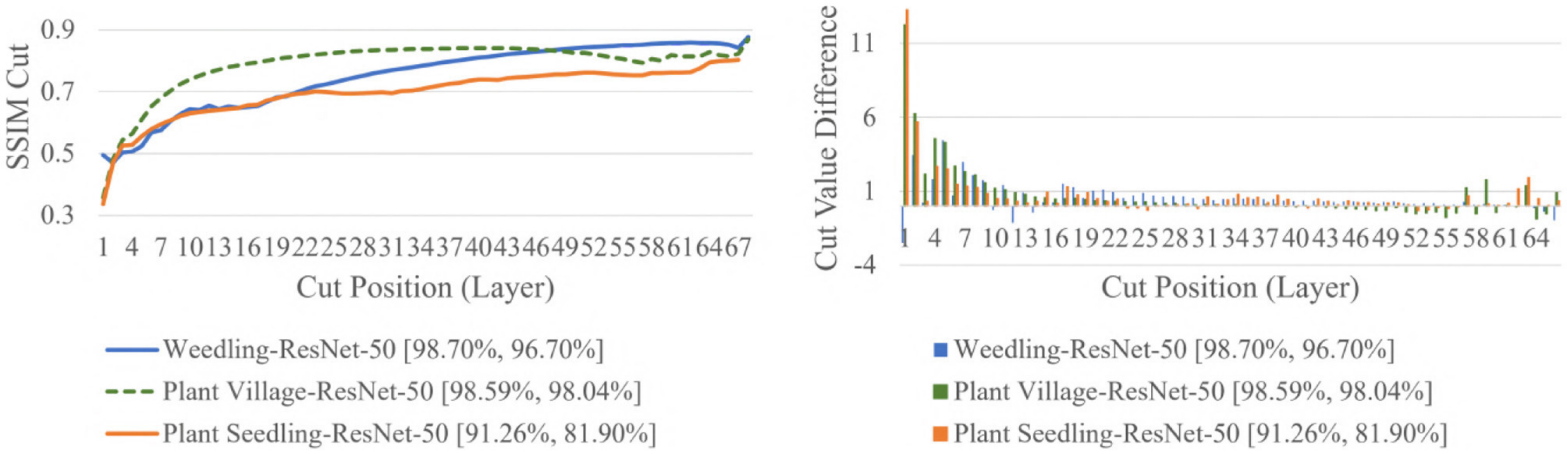
Comparison of the Cut Position (Layer) VS SSIM cut curve, and Cut Value Difference for the ResNet-50 models for different datasets. The value in the legend of the chart indicate the training and testing accuracy of the model.

The SSIM cut curve resembles the “elbow method,” commonly used in cluster analysis (Ketchen and Shook, 1996) to choose the number of clusters that optimize the clustering cost. For the SSIM cut curve, the elbow of the curve is a point where moving the cut position more to the right no longer improves the SSIM cut value significantly. Figure 4 shows the SSIM cut curves of ResNet-50 for different datasets. Initially, every SSIM cut curve shows a sharp positive slope, which indicates the feature visualizations for the initial layers are very dissimilar from the rest of the layers. The slope becomes flatter with the increase in cut position. Thus, in a model with many convolutional layers, the feature visualizations obtained from the shallow layers are more diverse than those from the deeper layers. Furthermore, the diversity of the feature visualizations at a deeper layer is larger in a balanced model compared to those in an overfit model (**RQ2**).

To evaluate whether the SSIM cut curve's elbow point could be used as a guide for selecting the depth of the model, we examined the performances of truncated ResNet-50 (i.e., 2-Conv-ResNet) for the same datasets. We observed that (Table 1) 2-Conv-ResNet achieved similar performance when compared with ResNet-50 for the Weedling dataset and even better performance for the Plant Village dataset. For the Seedling dataset, both the ResNet-50 and 2-Conv-ResNet remained overfit.

**Figure 5:**
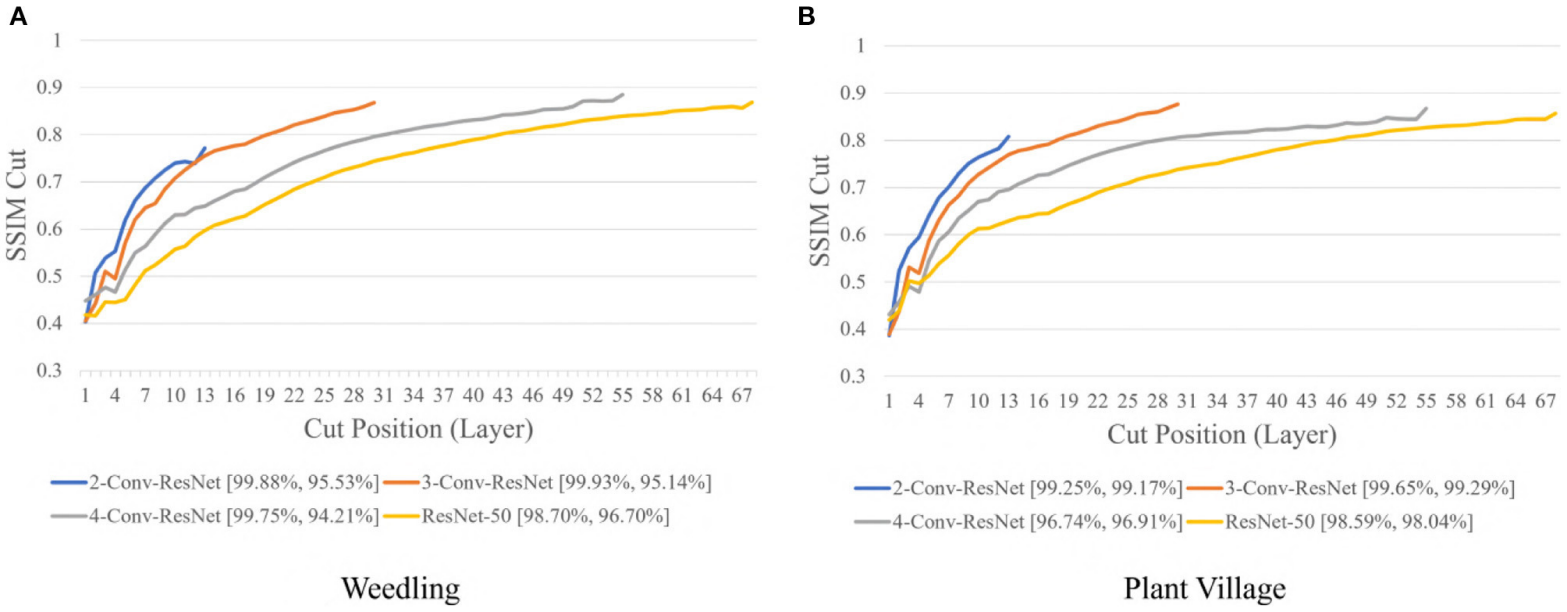
Comparison of the Cut Position (Layer) VS SSIM cut curve for ResNet models for **(A)** Weedling and **(B)** Plant Village dataset for Epoch (Best).

Next, we varied the number of blocks (see Section 2.5) in the ResNet-50 model to investigate the relation between the depth of a model and the performance of the model and also to see whether SSIM cut curve can help select the model depth more precisely. From Figure 5, we can see that the 2-Conv-ResNet and 3-Conv-ResNet have a sharper positive slope than 4-Conv-ResNet and ResNet-50 for both datasets. A flat SSIM cut curve indicates that the convolutional layers are learning less diverse features, indicating that a higher depth model will not always perform better. Table 1 supports this observation as compared to the larger 4-Conv-ResNet and ResNet-50; the 2-Conv-ResNet and 3-Conv-ResNet performed similarly for the Weedling dataset and better for the Plant Village dataset.

**Figure 6:**
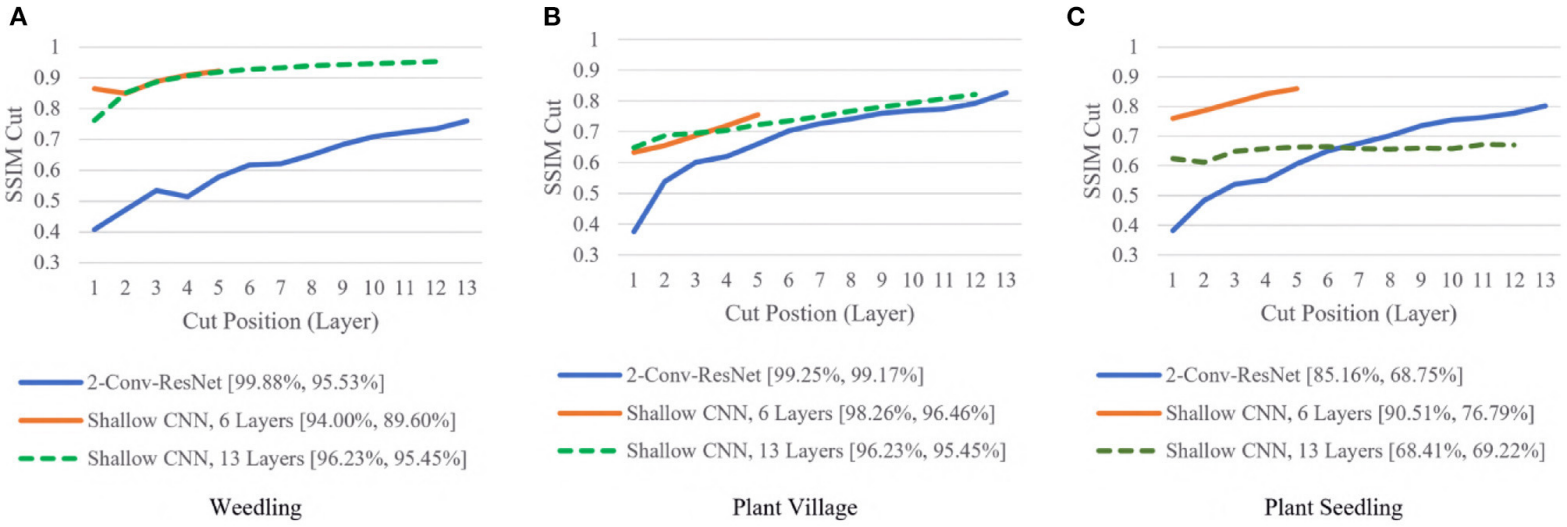
Comparison of the Cut Position (Layer) VS SSIM cut curve for the 2-Conv-ResNet, Shallow CNN, 6 Layers, and Shallow CNN, 13 Layers for different datasets. The value in the legend of the chart indicate the training and testing accuracy of the model. **(A)** Weedling. **(B)** Plant village. **(C)** Plant seedling.

To examine whether shallow models could achieve high performances, we compared the SSIM cut curve for the 2-Conv-ResNet, Shallow CNN with 6 Layers, and Shallow CNN with 13 layers (Figure 6).

**Figure 7:**
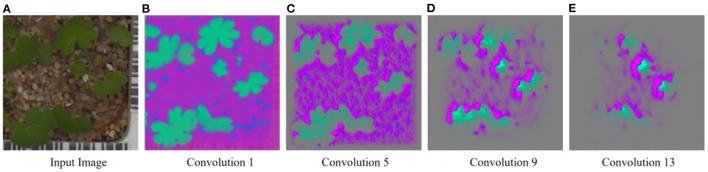
GBP visualization of the convolutional layers of the Small-flowered Cranesbill of the Plant Seedling dataset for Shallow CNN, 13 Layers. **(A)** Input image. **(B)** Convolution 1. **(C)** Convolution 5. **(D)** Convolution 9. **(E)** Convolution 13.

For the Weedling and Plant Village datasets, the Shallow CNN models achieved comparable performance to the ResNet-50 models. Furthermore, the Shallow CNN models with 6 layers performed similarly to CNN models with 13 layers. We observed a steady increase in the SSIM cut value in both cases. The Shallow CNN with 13 layers performed poorly for the Seedling dataset and relied on the background features (Figure 7). The Shallow CNN with 6 layers was overfit, but its training and test accuracy were higher than Shallow CNN with 13 layers.

**Figure 8:**
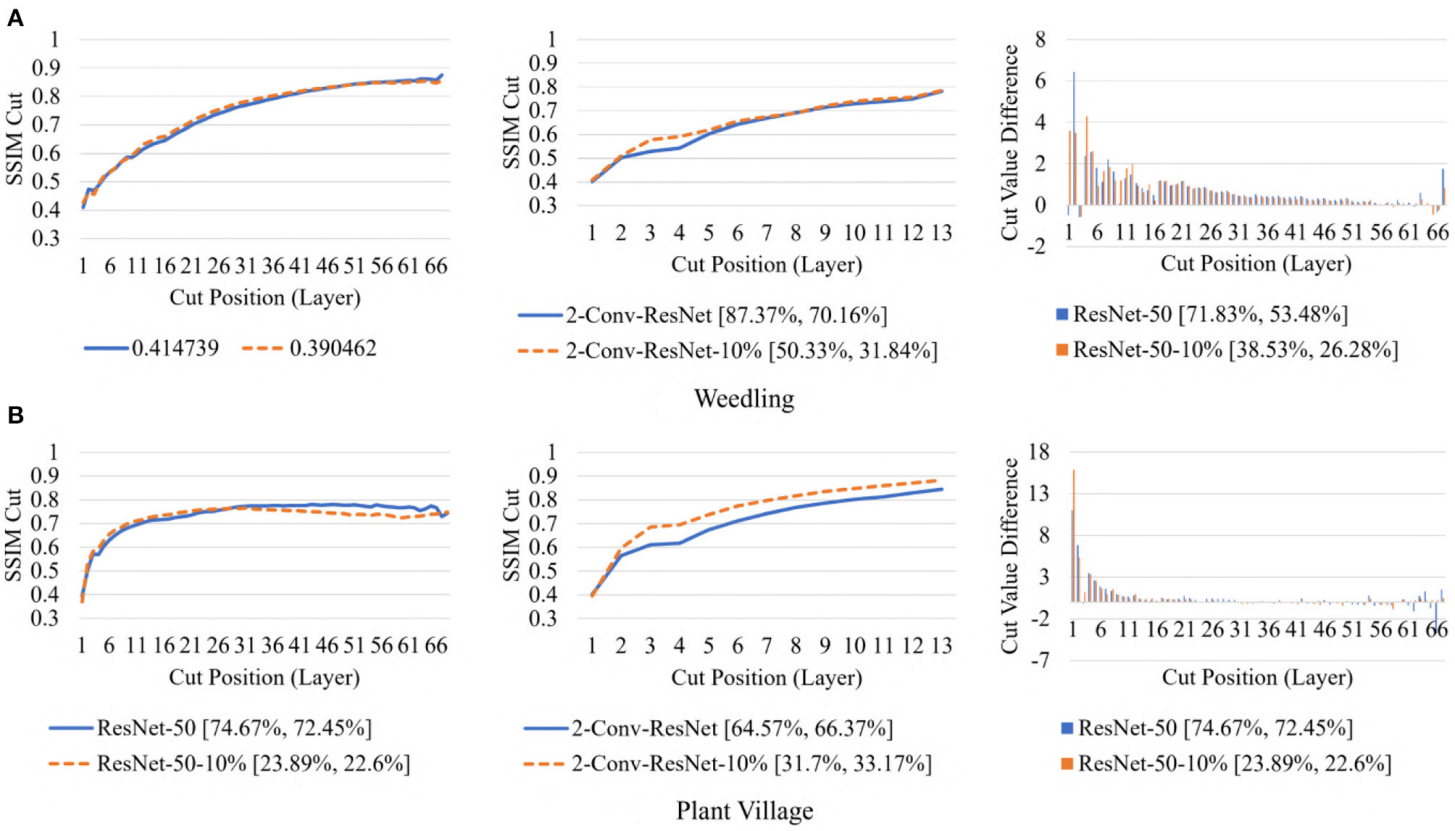
Comparison of the Cut Position (Layer) VS SSIM cut curve for the ResNet-50, ResNet-50-10%, 2-Conv-ResNet, and 2-Conv-ResNet-10% models, and Cut Value Difference for the ResNet-50, ResNet-50-10% for different datasets. The value in the legend of the chart indicate the training and testing accuracy of the model. **(A)** Weedling. **(B)** Plant village.

**Figure 9:**
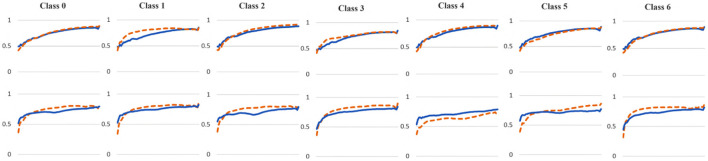
Comparison of the Cut Position (Layer) VS SSIM cut curve for different classes of the ResNet-50 (blue), and ResNet-50-10% (orange) models for (top row) Weedling and (bottom row) Plant Village dataset.

To examine the diversity of the feature visualizations between balanced and overfit models, we compared the SSIM cut curve of ResNet-50, ResNet-50-10%, 2-Conv-ResNet, and 2-Conv-ResNet-10% models for the Weedling and Plant Village dataset (Figure 8). ResNet-50 and 2-Conv-ResNet models were balanced for both datasets, and ResNet-50-10%, and 2-Conv-ResNet-10% models were overfit. In both cases, the SSIM curve of the overfit model had smaller initial SSIM cut values, which increased more sharply than in the balanced models. This can be observed better using the cut value difference plot. The decrease in the cut value difference was sharper for the overfit models for both datasets. This observation indicates that an overfit model may cease extracting new features earlier than a balanced model. A similar trend can also be seen for the per class analysis in Figure 9.

### 3.3. Model Selection Using SSIM Cut Curve

This section discusses whether the SSIM cut curve could be leveraged to select an appropriate ResNet-50 model. An ideal situation would be to check all the SSIM cut curves and select the one that learns over all the layers, i.e., where the SSIM cut curve is constantly rising. However, computing the SSIM cut curves for all the models (2-Conv-ResNet, 3-Conv-ResNet, 4-Conv-ResNet, ResNet-50) is infeasible due to the huge amount of time it requires to train these models. For example, it took 72.81 h and 7.80 h to train ResNet-50 for 100 epochs using the Weedling and Plant Village datasets. However, if we can predict the appropriate depth by examining these models only at their first training epochs, we can reduce the time for selecting an appropriate model. This idea would only work if the SSIM cut curves for various models at the first epoch and the best epoch maintained the same shape and relative ordering of the curves.

**Figure 10:**
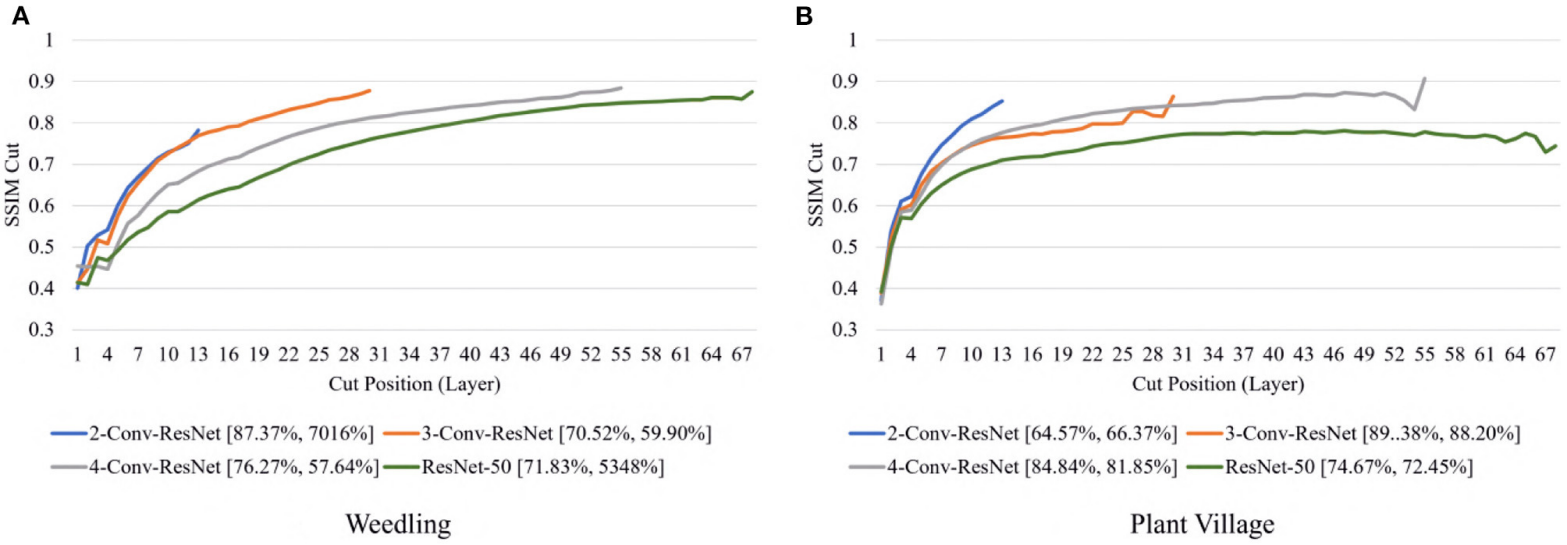
Comparison of the Cut Position (Layer) VS SSIM cut curve for ResNet models for **(A)** Weedling and **(B)** Plant Village dataset at Epoch (1). The value in the legend of the chart indicate the training and testing accuracy of the model.

The rapid increase in the SSIM cut curve indicates that the feature maps are extracting new and diverse features. The curve's saturation indicates that the feature maps may have stopped extracting additional features or learning very subtle features. To select a preferred depth of a model from the SSIM cut curve, we should consider the layers as long as the cut curve is rising, i.e., cut value differences are large. Figure 10 illustrates the SSIM cut curves for different models at the first epoch, which have a shape similar to the SSIM cut curves computed from the best epoch, e.g., see Figure 6. To precisely find the desired model depth, we can use the cut value difference curve and the SSIM cut curve. For the Weedling dataset, in Figure 12A, the change of cut value difference is much less after layer 31, which indicates that the rest of the layers might be redundant. A model with around 31 layers is likely to be able to replicate the performance of the ResNet-50 model. From Figures 6, 10, we can see that the SSIM cut curve of the 3-Conv-ResNet is consistently rising, with around 31 layers. From both figures, we can see that the change of the SSIM cut values of the 4-Conv-ResNet in later layers is much smaller, showing that the additional depth of the 4-Conv-ResNet fails to help learn additional features. The SSIM cut curve of the 2-Conv-ResNet always increases, indicating that the feature maps are still learning features, and more layers can help learn additional features. Based on these observations, we can say that the 3-Conv-ResNet is the preferred model for the Weedling dataset.

For the Plant Village dataset, the cut value difference of Figure 12B suggests that a model with around 16 layers should be sufficient to perform the task. In Figures 6, 10, the increasing SSIM cut curve of the 2-Conv-ResNet and 3-Conv-ResNet also supports these models being chosen as the preferred model. From Table 1, it is evident that the 3-Conv-ResNet outperforms all the models. So, the SSIM cut curve can help us choose the depth of a model.

**Figure 11:**
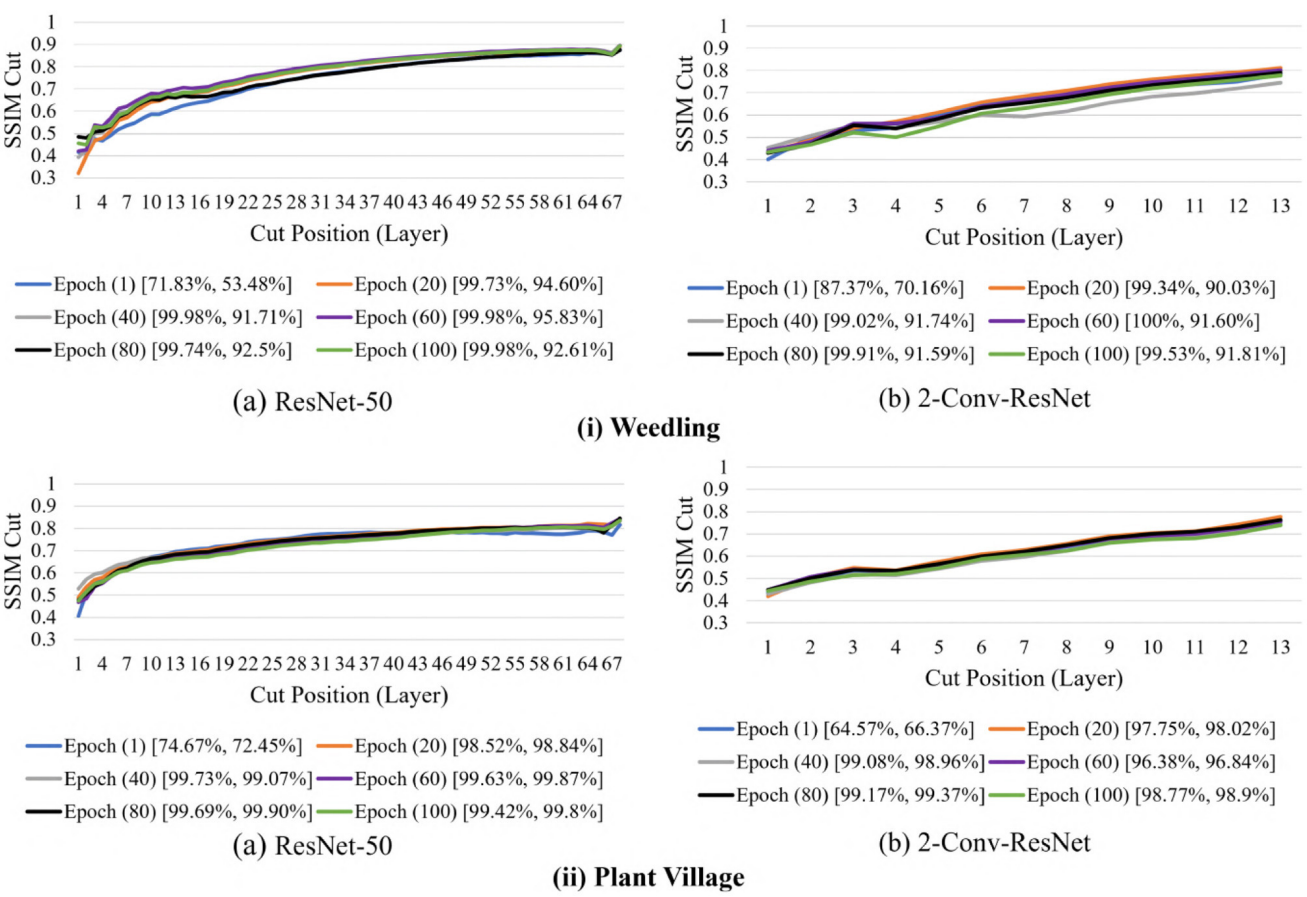
Comparison of the Cut Position (Layer) VS SSIM cut for different epochs of **(i)** Weedling and **(ii)** Plant Village dataset using (a) ResNet-50 and (b) 2-Conv-ResNet. The value in the legend of the chart indicate the training and testing accuracy of the model.

We now examine the behavior of the SSIM cut curve over various epochs. Figure 11 shows the SSIM cut curve of ResNet-50 and 2-Conv-ResNet models for Weedling and Plant Village datasets at different epochs. From Figure 11, we can see that the SSIM cut curves are similar at different epochs for a model, and the pattern is consistent for both models and both datasets. For both datasets, the SSIM cut curve of the ResNet-50 model suggests an earlier elbow point, whereas the 2-Conv-ResNet shows a steady increase. The stability of the SSIM cut at various epochs gives further evidence that relying on the first training epoch would be sufficient for the SSIM cut curve based model selection.

**Figure 12:**
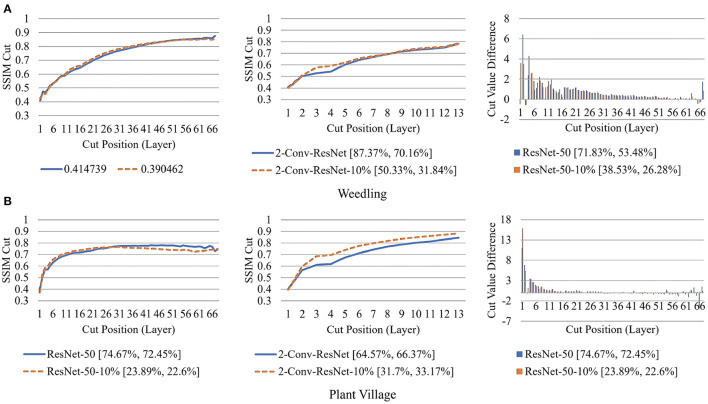
Comparison of the Cut Position (Layer) VS SSIM cut curve for the ResNet-50, ResNet-50-10%, 2-Conv-ResNet, and 2-Conv-ResNet-10% models, and Cut Value Difference for the ResNet-50, ResNet-50-10% for different datasets at Epoch (1). The value in the legend of the chart indicate the training and testing accuracy of the model. **(A)** Weedling. **(B)** Plant village.

Next, in Figure 12, we compared the SSIM cut curves for the first epoch of ResNet-50, ResNet-50-10%, 2-Conv-ResNet, and 2-Conv-ResNet-10% models along with their cut value difference plots for the ResNet-50 and ResNet-50-10%. From Figures 8, 12, it is evident that shape and relative ordering of the SSIM-cut curves obtained at the first epoch (Figure 12) is consistent with the model's best epoch (Figure 8).

Since the empirical results show that the SSIM cut curve follows the same trend throughout the training of a CNN, we can choose the preferred model depth by examining the SSIM cut curves for various models at the beginning of their training. So, the SSIM cut curve of the feature map visualizations may help detect potential underfitting or overfitting in the CNN models and allow us to select a balanced model (**RQ3**).

### 3.4. Early Stopping VS SSIM Cut Curve

There are several ways to avoid overfitting, and early stopping is one of them (Ying, 2019). Early stopping is a form of regularization that is used during training in iterative methods to select how long a model is going to be trained (Girosi et al., 1995; Prechelt, 1998). In early stopping, it is possible that the learning of the model is stopped before it is fully optimized (Caruana et al., 2000), which may prevent the model from making accurate predictions. Early stopping is insensitive to the capacity of the model. As a result, the training of the smaller models can be stopped before it is optimized (Caruana et al., 2000).

The advantage of the SSIM cut curve is that it helps select an optimized model. By looking at the SSIM cut of models of various depth, we propose the depth that is likely to provide better accuracy. For a fixed model, the shape of the SSIM cut curve remains similar whether we stop early or not (see Figures 6, 10). Therefore, we look at the SSIM cut curves of models built after the first epoch to expedite the process.

Note that early stopping may be used while training the model to expedite the model selection by generating SSIM cut curves. However, here we only used one training epoch to generate the curves. Once we select an appropriate model by examining the generated SSIM cut curves, we do not use any early stopping criteria on the selected model.

### 3.5. Summary

In summary, our experimental results suggest that the extraction of features of a deep learning model depends on the capacity of the model (**RQ1**). Our analysis of the SSIM curve shows that the GBP visualizations of the initial convolutional layers of a model are much more diverse than the GBP visualizations for the deeper layers (**RQ2**). Furthermore, the rate of change and the SSIM cut curve's elbow point can be used for model selection (**RQ3**). Since the SSIM cut curve is consistent for a model throughout different training epochs, we can use it to choose a model depth at an early training stage. This can save a lot of time in a traditional approach that compares models after fully training them.

### 3.6. Testing With Segmented Images

Table 2 illustrates the accuracy of different models on the Weedling and Plant Village dataset for segmented images. For segmentation, we retained all the green pixels in the image and marked the rest of the pixels as black (see Supplementary Material for examples of segmented images). Finally, we ended up with images where only the leaf was present. Next, we used the pre-trained models on the segmented images to calculate the accuracy.

**Table 2:** Performances of ResNet-50 model with different block for various datasets with segmented images.

	**Epoch (best)**		**Epoch (1)**	
	**Training dataset (%)**	**Testing dataset (%)**	**Training dataset (%)**	**Testing dataset (%)**
**Weedling**				
ResNet-50	58.87	53.61	14.01	11.99
ResNet-50-10%	41.95	35.49	28.43	25.60
2-Conv-ResNet	33.56	24.74	29.39	25.88
2-Conv-ResNet-10%	27.61	18.66	27.82	17.26
3-Conv-ResNet	**65.50**	**66.64**	14.08	12.08
4-Conv-ResNet	30.77	23.38	13.97	11.97
**Plant village**				
ResNet-50	28.00	23.68	10.60	10.50
ResNet-50-10%	31.45	27.80	10.08	11.54
2-Conv-ResNet	40.78	34.71	28.66	25.35
2-Conv-ResNet-10%	24.03	23.37	28.66	25.35
3-Conv-ResNet	**61.72**	**62.94**	30.94	26.52
4-Conv-ResNet	43.90	38.09	21.48	20.56

*Bold values represent the highest classification accuracy*.

From Table 2, we can see that the 3-Conv-ResNet has higher classification accuracy than other models for both Weedling and Plant Village dataset, which implies that the models are more focused on the leaf features than the background features. Also, the low accuracy of the ResNet-50 model indicates background features may more influence it than the 3-Conv-ResNet model. Comparing the accuracy of Epoch (1) to the accuracy of Epoch (Best), we can see that as the training progresses, the models tend to focus more on the leaf features than the background features. However, the results of such experiments can be limited by the quality of the segmented images.

## 4. Conclusion

In this article, we explained the overfitting in a CNN model for plant phenotyping by visualizing the intermediate layers' learning. We used guided backpropagation to visualize the learning of the intermediate layer of different CNN models. We used four different models on three different plant classification datasets. We proposed a novel SSIM cut based analysis to measure the similarity among the features learned in the intermediate layers of a CNN. Our experiments showed that the features extracted by a model depend on its capacity. Our SSIM cut curve revealed that in a more complex model, the shallow layers learn more diverse features as compared to the deeper layers and that a more distinct transition between these regimes is noticeable for overfit models. The SSIM cut curve method can help detect a potential overfit condition or inform a practitioner that a shallower model may be more appropriate for training with a particular dataset. We also showed the usage of the SSIM cut curve in selecting the model depth. It can help reduce a model's training time and resource as we can predict the required model depth at the beginning of training. We believe our study contributes to a better understanding of the behavior of overfit CNN models and provides new directions for creating metrics to detect and avoid model overfitting in plant phenotyping tasks.

Future works may further examine various facets of our SSIM cut curve based analysis. In our SSIM cut curve analysis, the elbow point may not always correspond to a sharp elbow or be identified unambiguously in practice, which is a commonly known limitation of elbow heuristics (Ketchen and Shook, 1996). We envision running a user study involving deep learning experts, where one can show the output of different models by hiding the model's label and recording their opinions to see whether a domain expert can detect an overfit model by only observing the GBP visualization of the intermediate layers. Due to the residual connection in the ResNet models, it might be possible to avoid overfitting and influence the similarity of the GBP visualizations of various layers. Hence it would be interesting to investigate the contribution of the residual connections in an overfit model's performance.

## Data Availability Statement

The original contributions presented in the study are included in the article/Supplementary Material, further inquiries can be directed to the corresponding author/s.

## Author Contributions

SM conceived of the presented idea and performed the computations. SM, DM, and IS developed the theory. DM and IS verified the analytical methods. MB, CB, and CH provided the Weedling dataset and the trained ResNet-50 model for analysis. All authors discussed the results and contributed to the final manuscript.

## Funding

This research was undertaken thank in part to funding from the Canada First Research Excellence Fund.

## Conflict of Interest

The authors declare that the research was conducted in the absence of any commercial or financial relationships that could be construed as a potential conflict of interest.

## Publisher's Note

All claims expressed in this article are solely those of the authors and do not necessarily represent those of their affiliated organizations, or those of the publisher, the editors and the reviewers. Any product that may be evaluated in this article, or claim that may be made by its manufacturer, is not guaranteed or endorsed by the publisher.
