# Supplementary material for: Leveraging Guided Backpropagation to Select Convolutional Neural Networks for Plant Classification
Source: Front Artif Intell. 2022 May 11;5:871162. doi: 10.3389/frai.2022.871162 (PMC9132261; doi:10.3389/frai.2022.871162)
Supplement: Supplementary file 1 [file Data_Sheet_1.PDF]

Visualizing Feature Maps for Model Selection in Convolutional Neural Networks

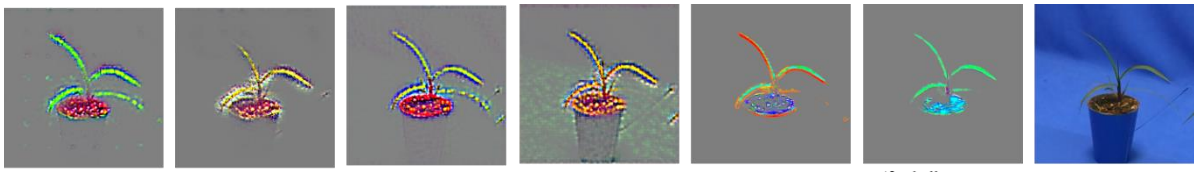

(a) ResNet-50 (b) ResNet-50-10% (c) 2-Conv-ResNet (d) 2-Conv-ResNet-10% (e) Shallow CNN, 6 Layers (f) Shallow CNN, 13 Layers (g) Input Image

(i) Barnyard Grass

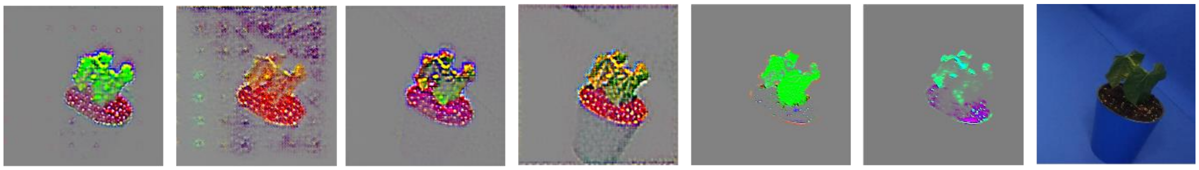

(a) ResNet-50 (b) ResNet-50-10% (c) 2-Conv-ResNet (d) 2-Conv-ResNet-10% (e) Shallow CNN, 6 Layers (f) Shallow CNN, 13 Layers (g) Input Image

(ii) Bean

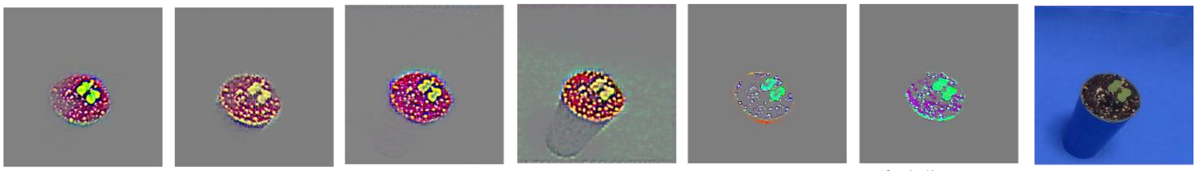

(a) ResNet-50 (b) ResNet-50-10% (c) 2-Conv-ResNet (d) 2-Conv-ResNet-10% (e) Shallow CNN, 6 Layers (f) Shallow CNN, 13 Layers (g) Input Image

(iii) Canola

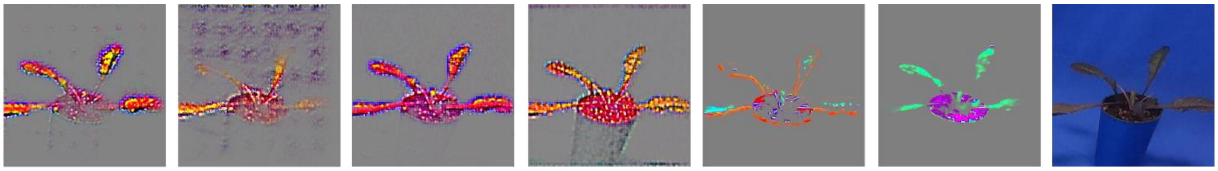

(a) ResNet-50 (b) ResNet-50-10% (c) 2-Conv-ResNet (d) 2-Conv-ResNet-10% (e) Shallow CNN, 6 Layers (f) Shallow CNN, 13 Layers (g) Input Image

(iv) Dandelion

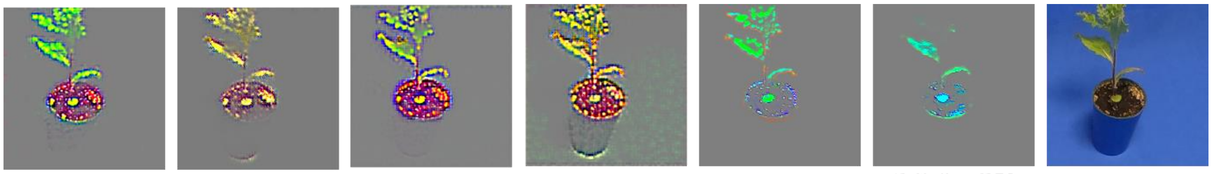

(a) ResNet-50 (b) ResNet-50-10% (c) 2-Conv-ResNet (d) 2-Conv-ResNet-10% (e) Shallow CNN, 6 Layers (f) Shallow CNN, 13 Layers (g) Input Image

(v) Soybean

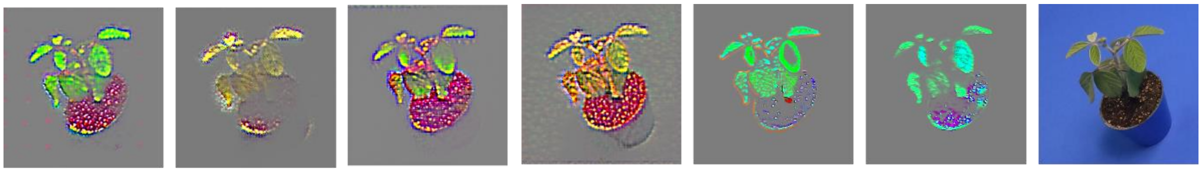

(a) ResNet-50 (b) ResNet-50-10% (c) 2-Conv-ResNet (d) 2-Conv-ResNet-10% (e) Shallow CNN, 6 Layers (f) Shallow CNN, 13 Layers (g) Input Image

(vi) Canada Thistle and Smartweeds

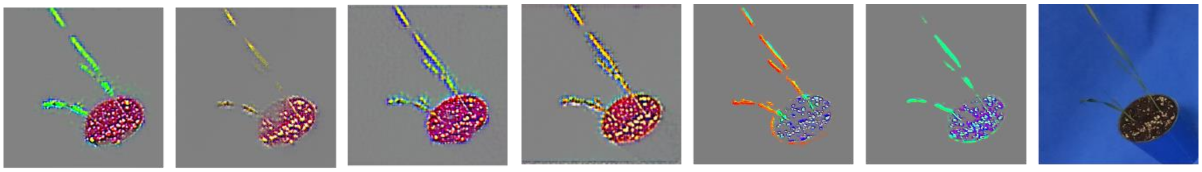

(a) ResNet-50 (b) ResNet-50-10% (c) 2-Conv-ResNet (d) 2-Conv-ResNet-10% (e) Shallow CNN, 6 Layers (f) Shallow CNN, 13 Layers (g) Input Image

(vii) Wheat

Supplementary Figure 1: GBP visualization of the last convolutional layer of different CNN models for different classes of the Weedling dataset.

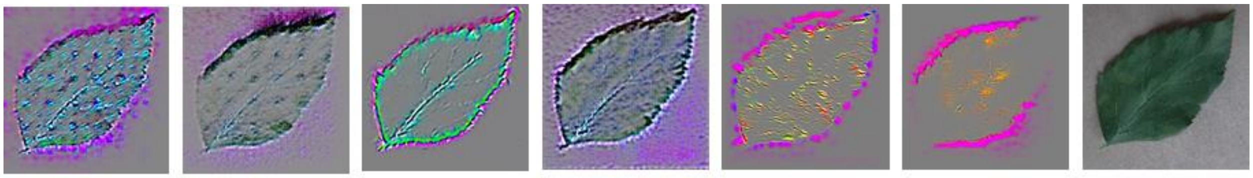

(a) ResNet-50 (b) ResNet-50-10% (c) 2-Conv-ResNet (d) 2-Conv-ResNet-10% (e) Shallow CNN, 6 Layers (f) Shallow CNN, 13 Layers (g) Input Image

(i) Apple

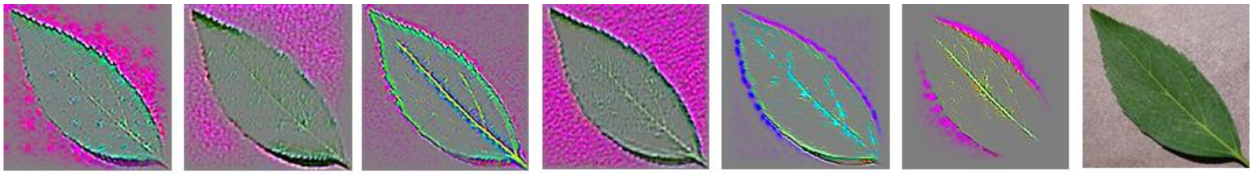

(a) ResNet-50 (b) ResNet-50-10% (c) 2-Conv-ResNet (d) 2-Conv-ResNet-10% (e) Shallow CNN, 6 Layers (f) Shallow CNN, 13 Layers (g) Input Image

(ii) Blueberry and Cherry

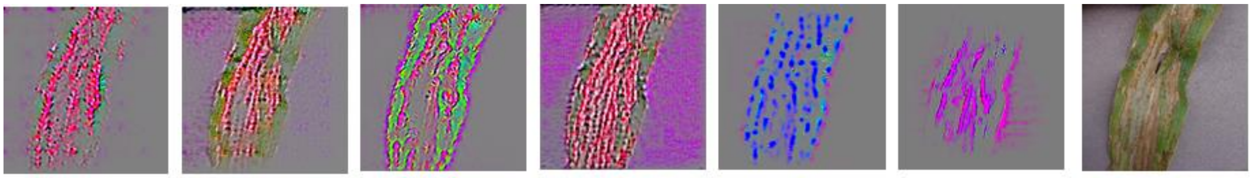

(a) ResNet-50 (b) ResNet-50-10% (c) 2-Conv-ResNet (d) 2-Conv-ResNet-10% (e) Shallow CNN, 6 Layers (f) Shallow CNN, 13 Layers (g) Input Image

(iii) Corn

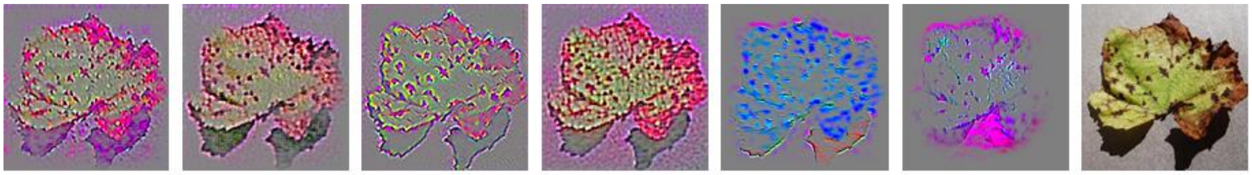

(a) ResNet-50 (b) ResNet-50-10% (c) 2-Conv-ResNet (d) 2-Conv-ResNet-10% (e) Shallow CNN, 6 Layers (f) Shallow CNN, 13 Layers (g) Input Image

(iv) Grape

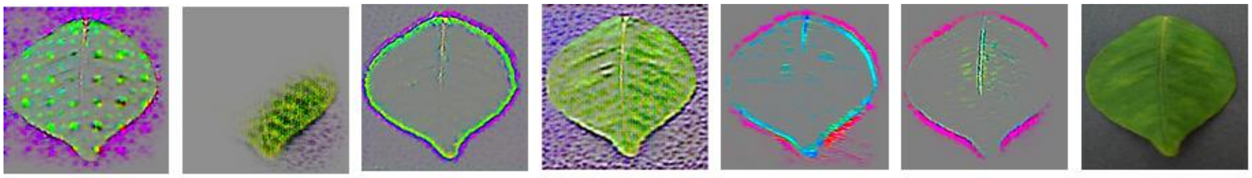

(a) ResNet-50 (b) ResNet-50-10% (c) 2-Conv-ResNet (d) 2-Conv-ResNet-10% (e) Shallow CNN, 6 Layers (f) Shallow CNN, 13 Layers (g) Input Image

(v) Orange

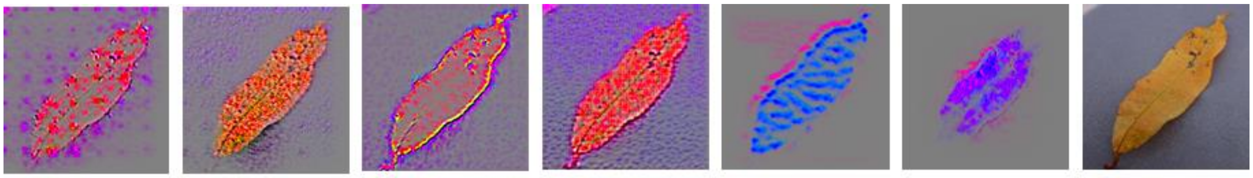

(a) ResNet-50 (b) ResNet-50-10% (c) 2-Conv-ResNet (d) 2-Conv-ResNet-10% (e) Shallow CNN, 6 Layers (f) Shallow CNN, 13 Layers (g) Input Image

(vi) Peach and Pepper

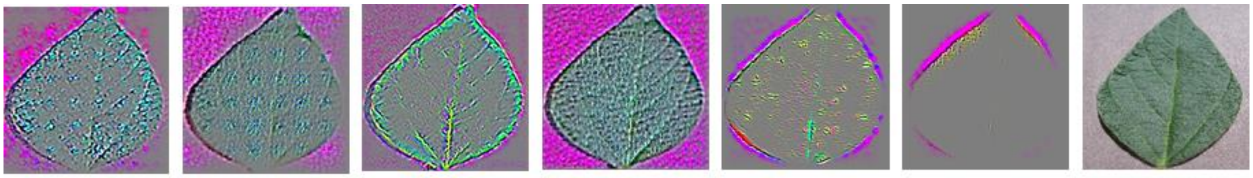

(a) ResNet-50 (b) ResNet-50-10% (c) 2-Conv-ResNet (d) 2-Conv-ResNet-10% (e) Shallow CNN, 6 Layers (f) Shallow CNN, 13 Layers (g) Input Image

(vii) Soybean

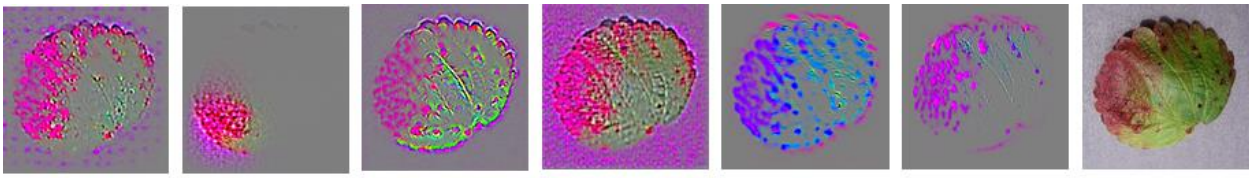

(a) ResNet-50 (b) ResNet-50-10% (c) 2-Conv-ResNet (d) 2-Conv-ResNet-10% (e) Shallow CNN, 6 Layers (f) Shallow CNN, 13 Layers (g) Input Image

(viii) Strawberry and Squash

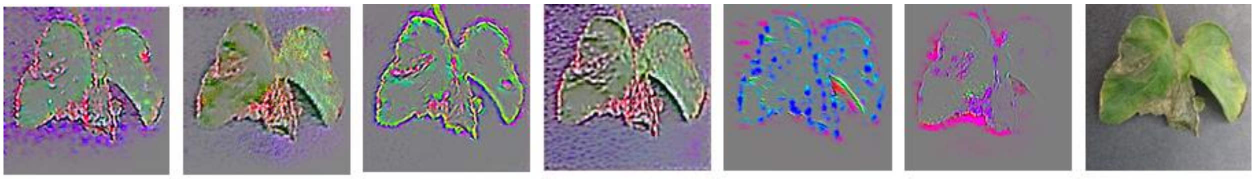

(a) ResNet-50 (b) ResNet-50-10% (c) 2-Conv-ResNet (d) 2-Conv-ResNet-10% (e) Shallow CNN, 6 Layers (f) Shallow CNN, 13 Layers (g) Input Image

(ix) Tomato

Supplementary Figure 2: GBP visualization of the last convolutional layer of different CNN models for different classes of the Plant Village dataset.

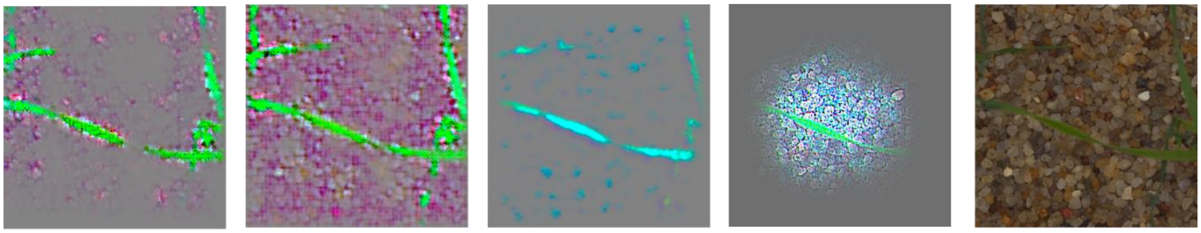

(a) ResNet-50 (c) 2-Conv-ResNet (e) Shallow CNN, 6 Layers (f) Shallow CNN, 13 Layers (g) Input Image

(i) Blackgrass

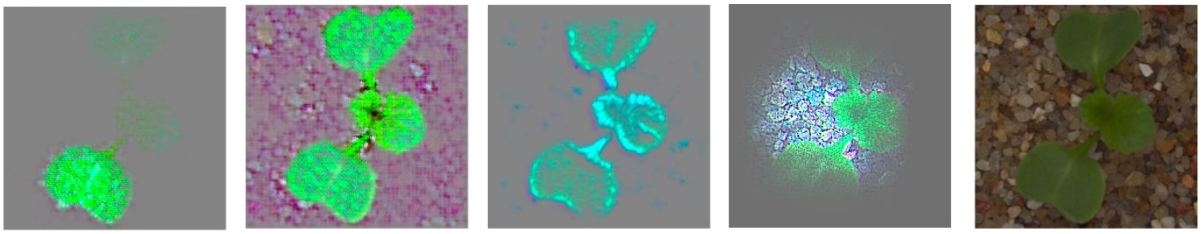

(a) ResNet-50 (c) 2-Conv-ResNet (e) Shallow CNN, 6 Layers (f) Shallow CNN, 13 Layers (g) Input Image

(ii) Charlock

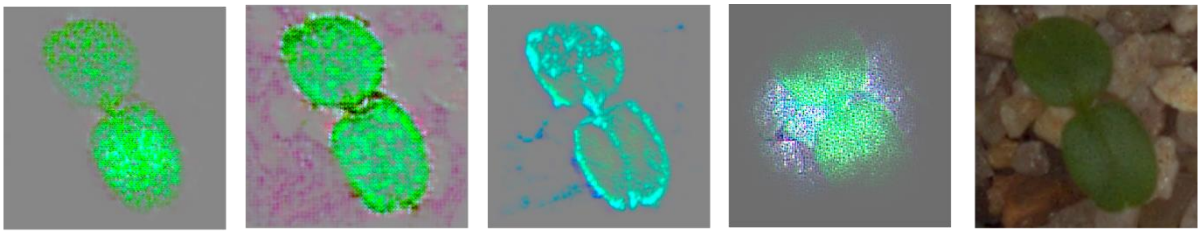

(a) ResNet-50 (c) 2-Conv-ResNet (e) Shallow CNN, 6 Layers (f) Shallow CNN, 13 Layers (g) Input Image

(iii) Cleavers

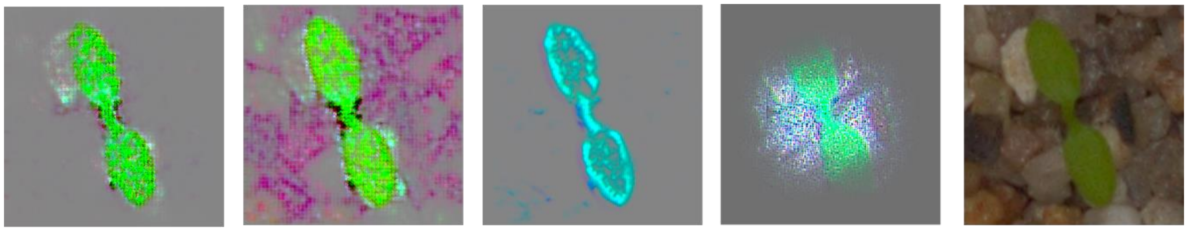

(a) ResNet-50 (c) 2-Conv-ResNet (e) Shallow CNN, 6 Layers (f) Shallow CNN, 13 Layers (g) Input Image

(iv) Chickweed

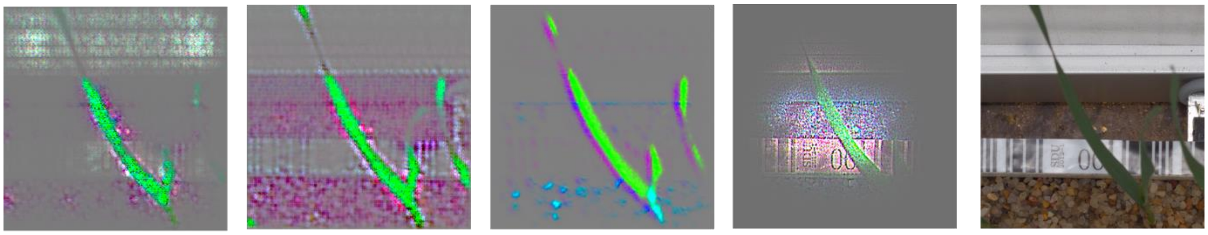

(a) ResNet-50 (c) 2-Conv-ResNet (e) Shallow CNN, 6 Layers (f) Shallow CNN, 13 Layers (g) Input Image

(v) Wheat

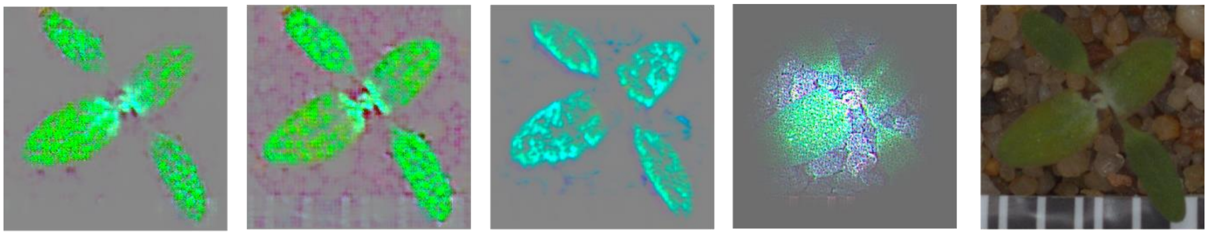

(a) ResNet-50 (c) 2-Conv-ResNet (e) Shallow CNN, 6 Layers (f) Shallow CNN, 13 Layers (g) Input Image

(vi) Fat hen

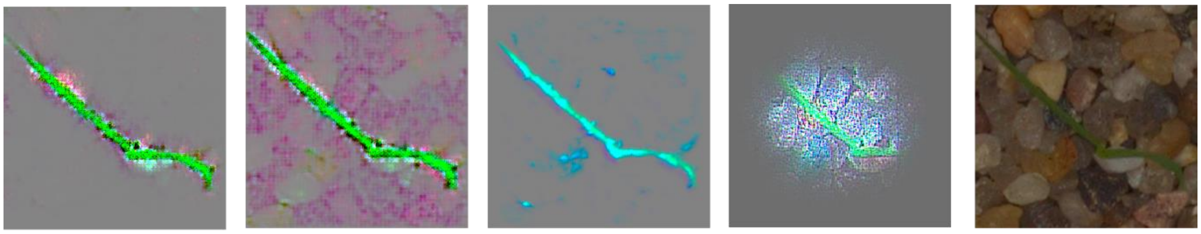

(a) ResNet-50 (c) 2-Conv-ResNet (e) Shallow CNN, 6 Layers (f) Shallow CNN, 13 Layers (g) Input Image

(vii) Loose silky-bent

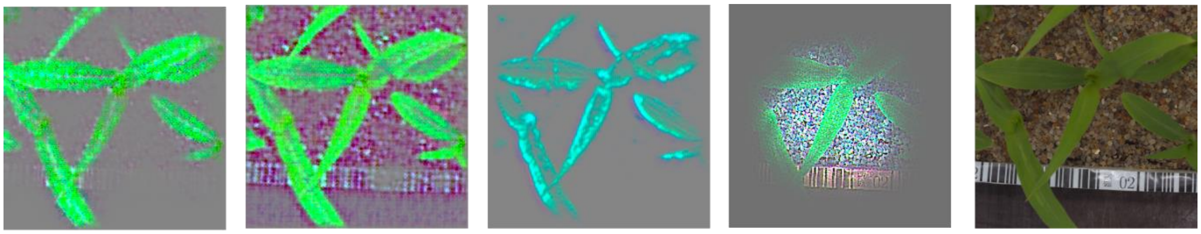

(a) ResNet-50 (c) 2-Conv-ResNet (e) Shallow CNN, 6 Layers (f) Shallow CNN, 13 Layers (g) Input Image

(viii) Maize

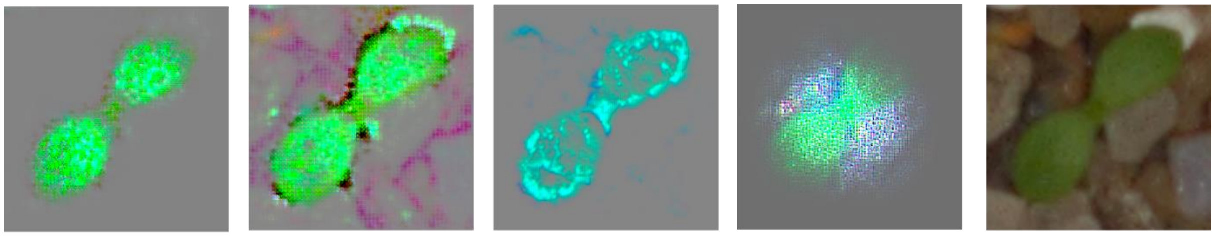

(a) ResNet-50 (c) 2-Conv-ResNet (e) Shallow CNN, 6 Layers (f) Shallow CNN, 13 Layers (g) Input Image

(ix) Scentless Mayweed

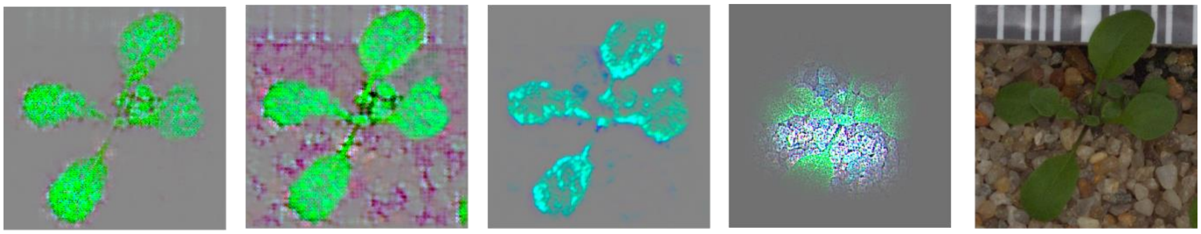

(a) ResNet-50 (c) 2-Conv-ResNet (e) Shallow CNN, 6 Layers (f) Shallow CNN, 13 Layers (g) Input Image

(x) Shepherds purse

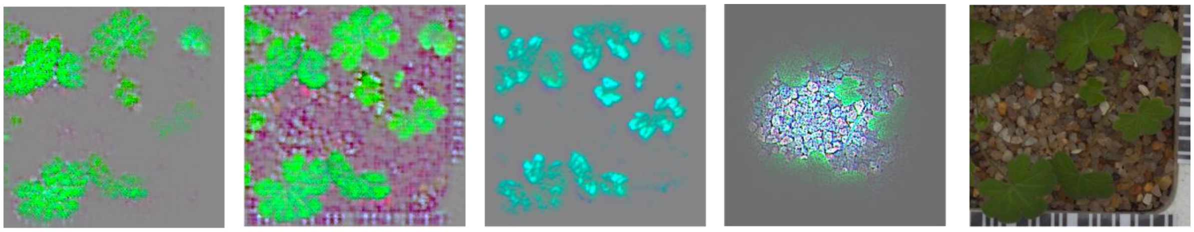

(a) ResNet-50 (c) 2-Conv-ResNet (e) Shallow CNN, 6 Layers (f) Shallow CNN, 13 Layers (g) Input Image

(xi) Small-flowered cranesbill

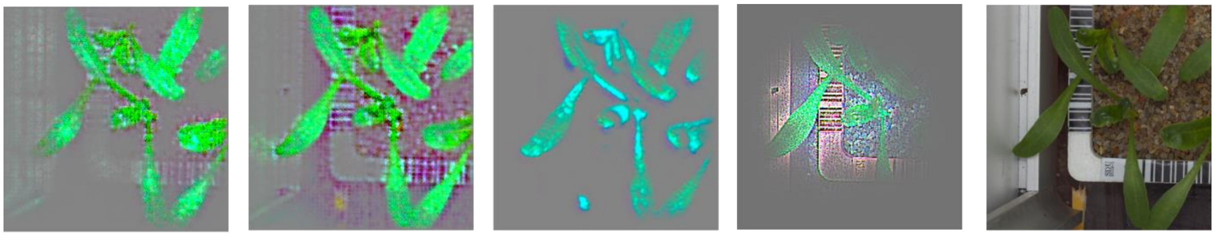

(a) ResNet-50 (c) 2-Conv-ResNet (e) Shallow CNN, 6 Layers (f) Shallow CNN, 13 Layers (g) Input Image

(xII) Sugar beet

Supplementary Figure 3: GBP visualization of the last convolutional layer of different CNN models for the different classes of the Plant Seedling dataset.

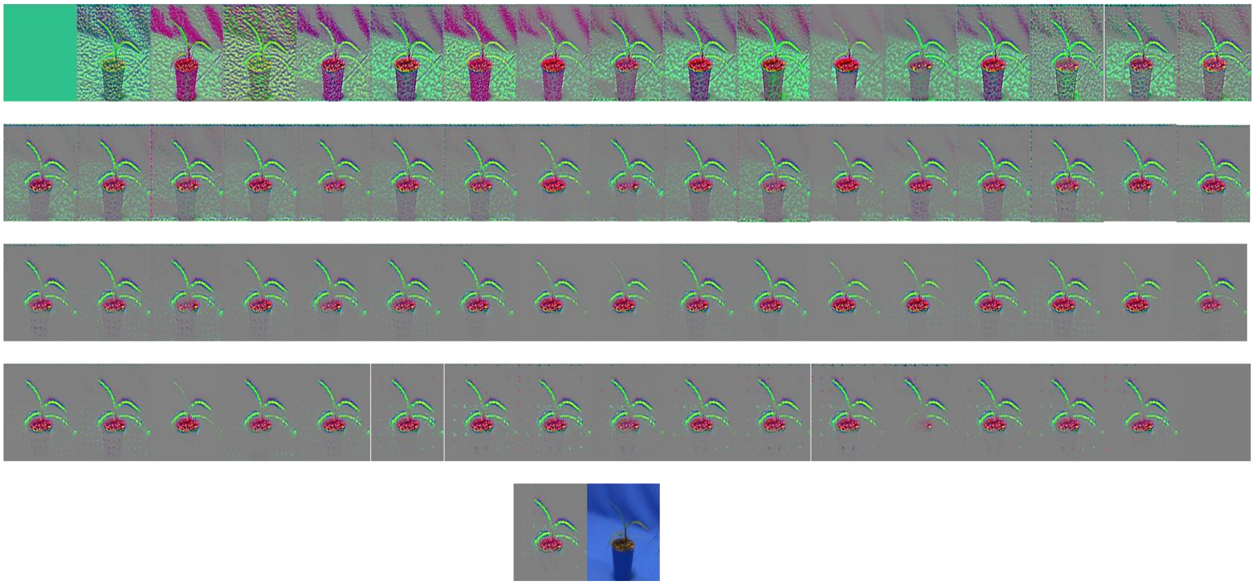

Supplementary Figure 4: Visualization of the learning of the intermediate layers of ResNet-50 using GBP for Barnyard Grass of the Weedling dataset.

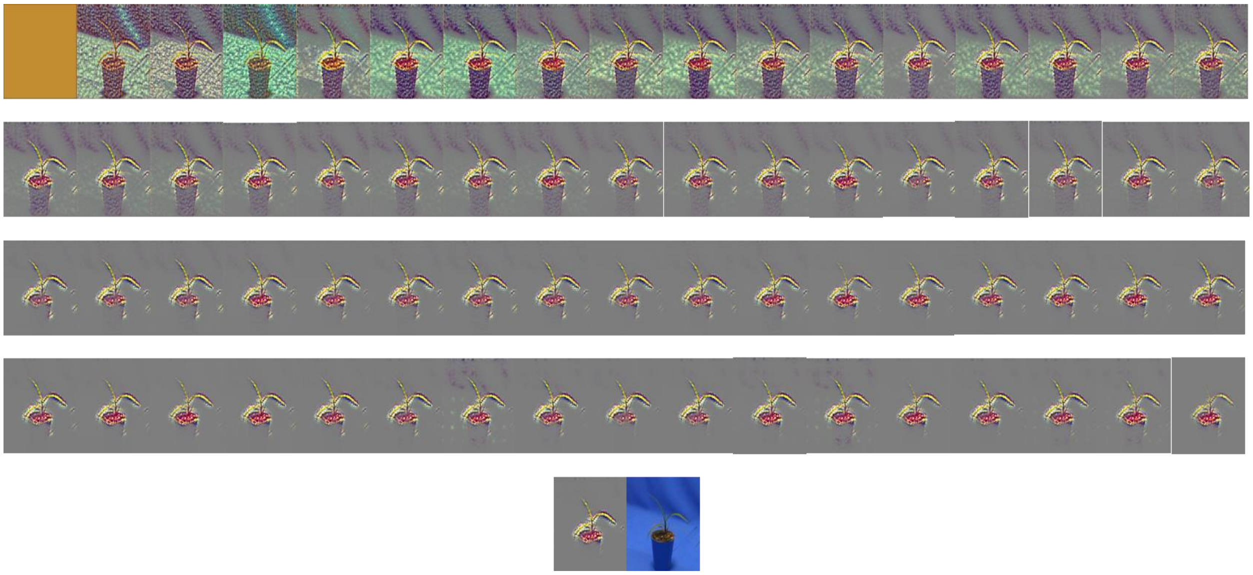

Supplementary Figure 5: Visualization of the learning of the intermediate layers of ResNet-50-10% using GBP for Barnyard Grass of the Weedling dataset.

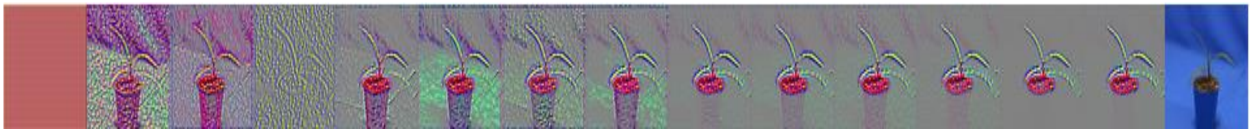

Supplementary Figure 6: Visualization of the learning of the intermediate layers of 2-Conv-ResNet using GBP for Barnyard Grass of the Weedling dataset.

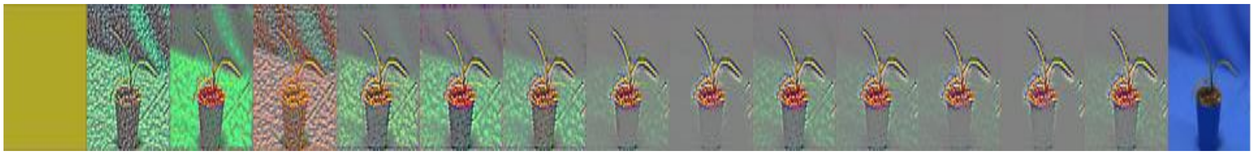

Supplementary Figure 7: Visualization of the learning of the intermediate layers of 2-Conv-ResNet-10% using GBP for Barnyard Grass of the Weedling dataset.

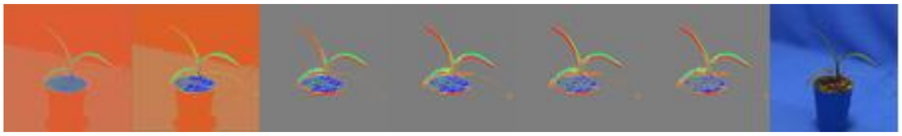

Supplementary Figure 8: Visualization of the learning of the intermediate layers of Shallow-CNN, 6 layers using GBP for Barnyard Grass of the Weedling dataset.

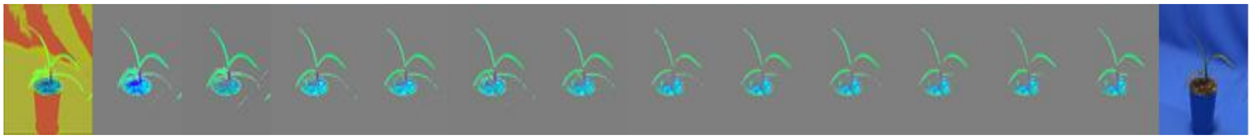

Supplementary Figure 9: Visualization of the learning of the intermediate layers of Shallow-CNN, 13 layers using GBP for Barnyard Grass of the Weedling dataset.

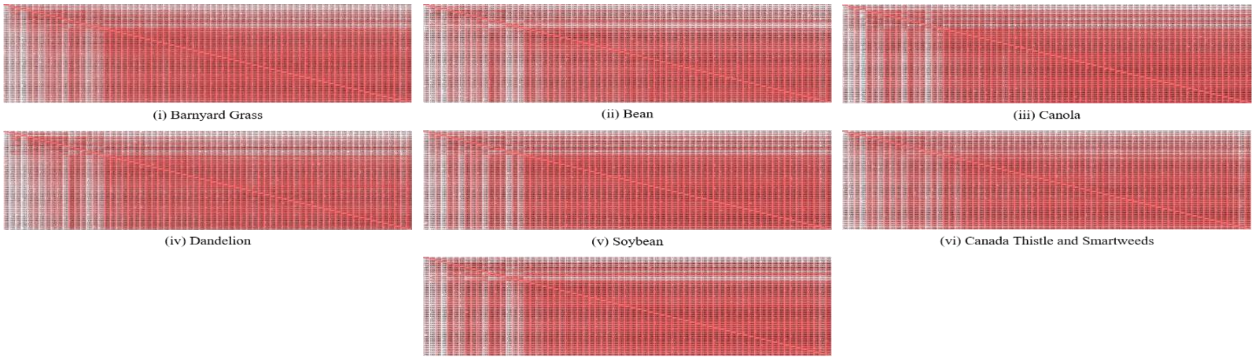

(i) Barnyard Grass (ii) Bean (iii) Canola

(iv) Dandelion (v) Soybean (vi) Canada Thistle and Smartweeds

(vii) Wheat

(a) ResNet-50

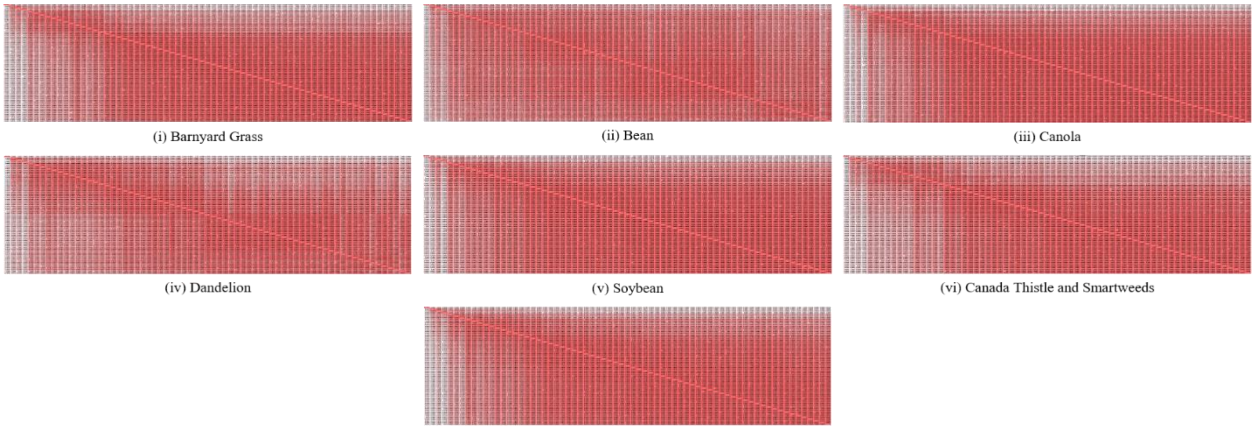

(i) Barnyard Grass (ii) Bean (iii) Canola

(iv) Dandelion (v) Soybean (vi) Canada Thistle and Smartweeds

(vii) Wheat

(b) ResNet-50-10%

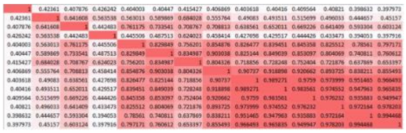
(i) Barnyard Grass

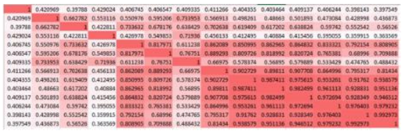
(ii) Bean

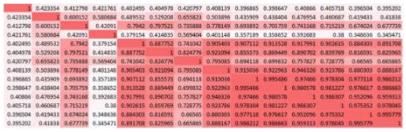
(iii) Canola

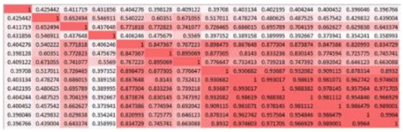
(iv) Dandelion

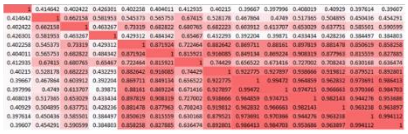
(v) Soybean

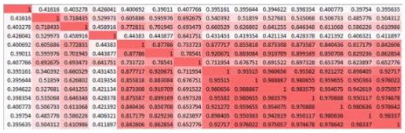
(vi) Canada Thistle and Smartweeds

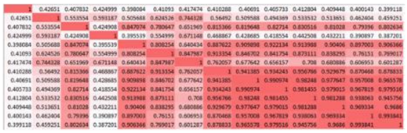
(vii) Wheat

(c) 2-Conv-ResNet

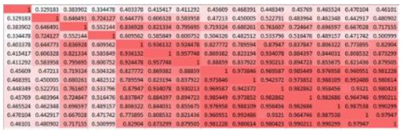
(i) Barnyard Grass

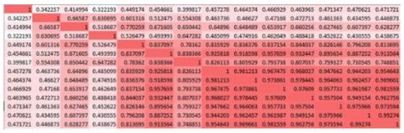
(ii) Bean

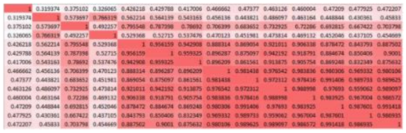
(iii) Canola

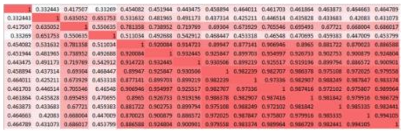
(iv) Dandelion

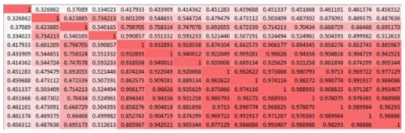
(v) Soybean

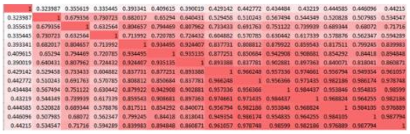
(vi) Canada Thistle and Smartweeds

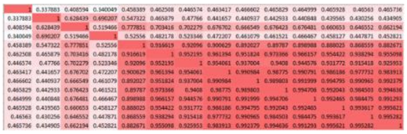
(vii) Wheat

(d) 2-Conv-ResNet-10%

1	0.968888	0.862261	0.85791	0.859287	0.86172
0.968888	1	0.858899	0.855433	0.853906	0.8558
0.862261	0.858899	1	0.982516	0.97551	0.974613
0.85791	0.855433	0.982516	1	0.987809	0.984907
0.859287	0.853906	0.97551	0.987809	1	0.991084
0.86172	0.8558	0.974613	0.984907	0.991084	1

(i) Barnyard Grass

1	0.935738	0.851856	0.850385	0.846922	0.845728
0.935738	1	0.905575	0.903982	0.897934	0.893935
0.851856	0.905575	1	0.98367	0.974166	0.967508
0.850385	0.903982	0.98367	1	0.979536	0.97754
0.846922	0.897934	0.974166	0.979536	1	0.986114
0.845728	0.893935	0.967508	0.97754	0.986114	1

(ii) Bean

1	0.964234	0.840764	0.842911	0.844213	0.848503
0.964234	1	0.824434	0.826602	0.826712	0.827868
0.840764	0.824434	1	0.985641	0.976963	0.971389
0.842911	0.826602	0.985641	1	0.987808	0.981532
0.844213	0.826712	0.976963	0.987808	1	0.989648
0.848503	0.827868	0.971389	0.981532	0.989648	1

(iii) Canola

1	0.968283	0.840693	0.846968	0.838454	0.844898
0.968283	1	0.842873	0.848425	0.840209	0.844692
0.840693	0.842873	1	0.966187	0.955779	0.953099
0.846968	0.848425	0.966187	1	0.975487	0.975076
0.838454	0.840209	0.955779	0.975487	1	0.983199
0.844898	0.844692	0.953099	0.975076	0.983199	1

(iv) Dandelion

1	0.938164	0.855906	0.855517	0.853136	0.851882
0.938164	1	0.893095	0.88974	0.88415	0.87799
0.855906	0.893095	1	0.983134	0.970841	0.960202
0.855517	0.88974	0.983134	1	0.98368	0.971555
0.853136	0.88415	0.970841	0.98368	1	0.982674
0.851882	0.87799	0.960202	0.971555	0.982674	1

(v) Soybean

1	0.914373	0.824087	0.831321	0.823245	0.819705
0.914373	1	0.851504	0.848104	0.844394	0.835137
0.824087	0.851504	1	0.968212	0.954261	0.937325
0.831321	0.848104	0.968212	1	0.972415	0.955009
0.823245	0.844394	0.954261	0.972415	1	0.9685
0.819705	0.835137	0.937325	0.955009	0.9685	1

(vi) Canada Thistle and Smartweeds

1	0.959073	0.843918	0.835654	0.834253	0.838359
0.959073	1	0.844005	0.835215	0.830199	0.830727
0.843918	0.844005	1	0.973908	0.961758	0.957357
0.835654	0.835215	0.973908	1	0.981283	0.976846
0.834253	0.830199	0.961758	0.981283	1	0.988287
0.838359	0.830727	0.957357	0.976846	0.988287	1

(vii) Wheat

(e) Shallow CNN, 6 Layers

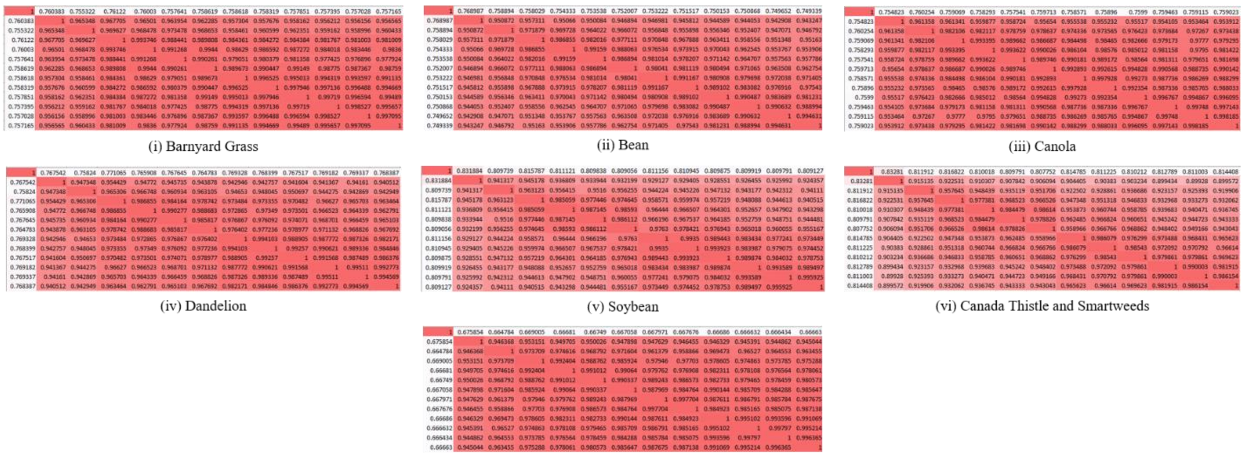

(i) Barnyard Grass (ii) Bean (iii) Canola (iv) Dandelion (v) Soybean (vi) Canada Thistle and Smartweeds (vii) Wheat

(f) Shallow CNN, 13 Layers

Supplementary Figure 10: SSIM between every pair of images generated with GBP for different classes of the Weedling dataset.

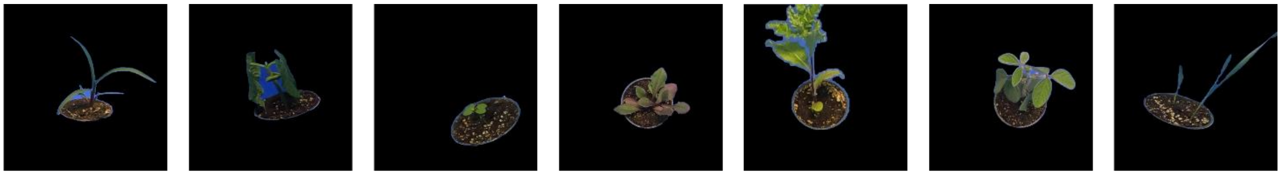

(i) Barnyard Grass (ii) Beans (iii) Canola (iv) Dandelion (v) Other Weed (vi) Soybean (vii) Wheat

(a) Weedling

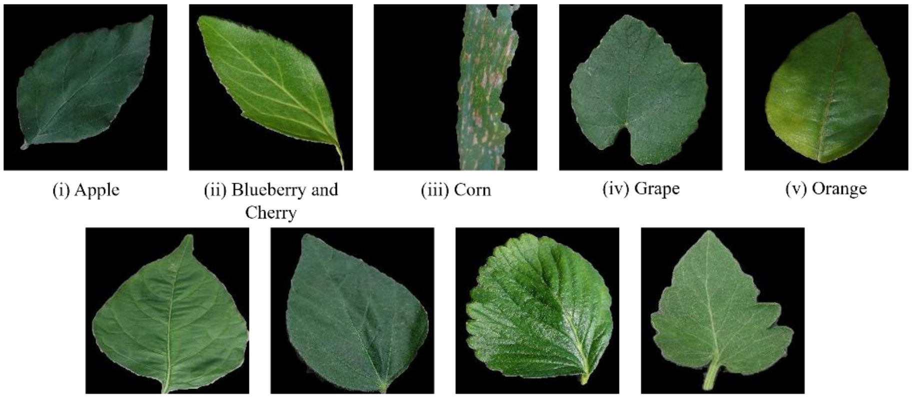

(i) Apple (ii) Blueberry and Cherry (iii) Corn (iv) Grape (v) Orange (vi) Peach and Pepper (vii) Soybean (viii) Strawberry and Squash (iv) Tomato

(b) Plant Village

Supplementary Figure 11: Example of segmented images for every class of the (a) Weedling and (b) Plant Village dataset.

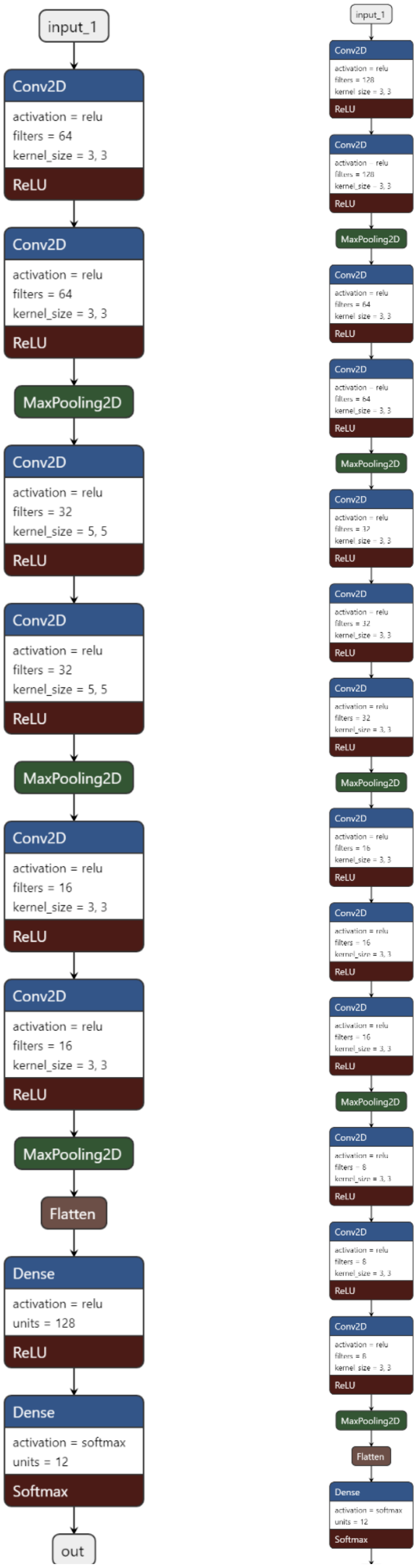

Supplementary Figure 12: Model Architecture of the Shallow CNN, 6 Layers (left), and Shallow CNN, 13 Layers (right)
